# Genome-Wide Profiling of DNA Methylation Reveals a Class of Normally Methylated CpG Island Promoters

**DOI:** 10.1371/journal.pgen.0030181

**Published:** 2007-10-26

**Authors:** Lanlan Shen, Yutaka Kondo, Yi Guo, Jiexin Zhang, Li Zhang, Saira Ahmed, Jingmin Shu, Xinli Chen, Robert A Waterland, Jean-Pierre J Issa

**Affiliations:** 1 Department of Leukemia, The University of Texas at M. D. Anderson Cancer Center, Houston, Texas, United States of America; 2 Aichi Cancer Center, Division of Molecular Oncology, Chikusa-Ku, Nagoya, Japan; 3 Biostatistics and Applied Biomathematics, The University of Texas at M. D. Anderson Cancer Center, Houston, Texas, United States of America; 4 Department of Pediatrics, Baylor College of Medicine, USDA Children's Nutrition Research Center, Houston, Texas, United States of America; Netherlands Cancer Institute, The Netherlands

## Abstract

The role of CpG island methylation in normal development and cell differentiation is of keen interest, but remains poorly understood. We performed comprehensive DNA methylation profiling of promoter regions in normal peripheral blood by methylated CpG island amplification in combination with microarrays. This technique allowed us to simultaneously determine the methylation status of 6,177 genes, 92% of which include dense CpG islands. Among these 5,549 autosomal genes with dense CpG island promoters, we have identified 4.0% genes that are nearly completely methylated in normal blood, providing another exception to the general rule that CpG island methylation in normal tissue is limited to X inactivation and imprinted genes. We examined seven genes in detail, including *ANKRD30A*, *FLJ40201*, *INSL6*, *SOHLH2*, *FTMT*, *C12orf12*, and *DPPA5*. Dense promoter CpG island methylation and gene silencing were found in normal tissues studied except testis and sperm. In both tissues, bisulfite cloning and sequencing identified cells carrying unmethylated alleles. Interestingly, hypomethylation of several genes was associated with gene activation in cancer. Furthermore, reactivation of silenced genes could be induced after treatment with a DNA demethylating agent or in a cell line lacking DNMT1 and/or DNMT3b. Sequence analysis identified five motifs significantly enriched in this class of genes, suggesting that *cis*-regulatory elements may facilitate preferential methylation at these promoter CpG islands. We have identified a group of non-X–linked bona fide promoter CpG islands that are densely methylated in normal somatic tissues, escape methylation in germline cells, and for which DNA methylation is a primary mechanism of tissue-specific gene silencing.

## Author Summary

About half of all human genes contain a CpG-rich region called a “CpG island” in the 5′ area, often encompassing the promoter and transcription start site of the associated gene. DNA methylation was initially suggested to control tissue-specific gene expression in mammalian cells, but most promoter region CpG islands were found to be unmethylated regardless of tissue specificity of expression. In this study, we discovered an exceptional subset of autosomal genes associated with dense promoter CpG islands that is methylated in normal tissues. We observed tissue-specific gene silencing correlated with hypermethylation in this class of genes, and provided evidence for a direct role of methylation in maintaining the silencing state. Furthermore, we identified five sequence motifs significantly enriched in this class of genes, suggesting the influence of *cis*-regulatory elements on the establishment and/or stability of DNA methylation. Together, these results provide important new insights into the role of CpG island methylation in normal development and differentiation.

## Introduction

CpG islands (CGIs) are discrete CpG-rich regions present in the promoters of 50%–70% of human genes [1]. DNA methylation within CGIs is associated with mitotically stable gene silencing (an epigenetic process). Such CGI methylation is involved physiologically in genomic imprinting [2] and X inactivation [3] and pathologically in developmental diseases [4] and cancer [5]. The role of CGI methylation in normal development, stem cell physiology, and differentiation, however, remains poorly understood [6].

Inhibition of DNA methylation can transform fibroblasts into muscle cells and other differentiated cells, suggesting that gene methylation regulates the process of differentiation [7]. However, support for this idea was dampened when CGIs were generally found to be unmethylated regardless of tissue-specific expression [8], and tissue-specific genes thought to be regulated by methylation were unaffected by demethylation in vivo [9]. It is now widely held that CGIs associated with both housekeeping and tissue-specific genes are unmethylated at any developmental stage, except when associated with certain imprinted genes and genes subject to X inactivation.

While most CGIs are unmethylated in normal tissues, there is increasing evidence that a few CGIs are in fact methylated in normal tissues independent of imprinting and X inactivation, and may play a role in the differentiation process through programmed expression of tissue-specific genes. At several genes, CpG island methylation has been correlated with transcriptional inactivation in normal cells. Most such correlations, however, involve intragenic CGIs rather than promoter CGIs [10,11]. A few promoter CGIs were found at which methylation correlates with transcription [12,13], but those contain a low density of CpG sites. For example, a recent study profiling genome-wide DNA methylation in three human chromosomes (Chr 6, 20, and 22) demonstrated that a small subset of promoter-region CGIs are methylated in various normal tissues, but all have a CpG density less than 10% [14]. That study also identified a considerable number of tissue-differentially methylated regions in CGIs, but these were preferentially located several kilobases away from the transcription start site of associated genes. Thus, the dogma that promoter-associated dense CGIs are unmethylated in normal tissues persists. A full appreciation of the role of CGI methylation in normal development awaits a careful high-throughput analysis of the process.

In this study, we compared differential methylation in normal tissues by genome-wide CGI methylation profiling and discovered non-X–linked bona fide promoter CGIs that are densely methylated in normal somatic tissues and escape methylation in germline cells. CGI hypermethylation at these gene promoters appears to be a primary mechanism of tissue-specific gene silencing.

## Results

### Genome-Wide Methylation Analysis by Methylated CpG Island Amplification in Combination with Microarray

We performed a comprehensive DNA methylation profiling of gene promoter regions in normal peripheral blood by methylated CpG island amplification (MCA) in combination with microarrays (MCAM). This procedure is described in Materials and Methods and outlined in Figure S1. Essentially, MCAM involves two steps. First, MCA [15] is used to enrich for methylated fragments. Second, labeling and cohybridization of MCA products to arrays enables comparison of locus-specific methylation between samples. We used promoter microarrays that contained 45- to 60-mer oligonucleotide probes covering from −1.0 kb to +0.3 kb relative to the transcription start sites of 18,000 human genes. Bioinformatic analysis predicted that 22,294 probes corresponding to 6,177 unique genes on the array would be informative when using SmaI/XmaI enzymes to generate methylated fragments up to 1 kb in size (see Materials and Methods). We also annotated all the SmaI/XmaI sites for CGIs and repetitive sequences. Among these informative genes on the arrays, gene promoters associated with dense-CGIs, sparse-CGIs and non-CGIs were 5,692 (92%), 318 (5%), and 167 (3%) respectively.

In an initial validation of MCAM, we compared in vitro fully methylated genomic DNA with DNA isolated from normal peripheral blood leukocytes (PBLs). As expected, the signal intensity of Cy5 (fully methylated) was high at most (87.1 %) informative probes (Figure S2A and S2B). The 12.9% of probes that did not show high signal in the positive control could be attributed to a PCR bias caused by the increased complexity of performing MCA on an artificially hypermethylated genome. Alternatively, nonsignificant signal could occur at probes with poor discriminative ability. Therefore, we estimated that MCAM technique has a false negative rate of less than 12.9%.

We compared the signal intensity of fluorescent probes between two independent hybridizations using MCA products processed at different times from the same normal PBL DNA sample, and found that the correlation between the two duplicates was 0.94, indicating that the technique is highly reproducible (Figure S2C).

In the cohybidization of MCA product from fully methylated DNA and normal PBL DNA, we observed probes showing high signal intensity in both channels (Figure S2B), suggesting genes hypermethylated in normal PBLs. Surprisingly, a subset of genes showed significantly higher signal in PBLs than in fully methylated DNA. This is possible because the relatively low number of hypermethylated regions in PBLs will amplify with higher efficiency compared with fully methylated DNA in the MCA reaction. We randomly selected 38 genes showing such high signal intensity in PBLs and analyzed them by a quantitative bisulfite pyrosequencing method. Of these, 17 showed dense (>70%) methylation in normal PBLs, six showed moderate (15% to 70%) methylation, and 15 showed low (<15%) methylation (Figure S3). Genes that were densely methylated in PBLs showed the highest signal intensity ratio (PBLs versus fully methylated DNA), with median ratio of 2.2; the median ratio in genes with moderate and low methylation was 1.5 and 0.7, respectively. Hence, a relatively high signal intensity (>3-fold of background) combined with a signal ratio ≥ 1.5 relative to in vitro-methylated DNA appears to identify hypermethylated loci in PBLs with 93% specificity and 74% sensitivity.

### Promoter Methylation in Normal Tissues

Using these criteria, we identified 455 genes methylated in normal PBLs (Table S1). 258 of these gene promoters were associated with dense-CGIs, 129 were associated with sparse-CGIs, and 68 were associated with non-CGIs. Thus, we estimate that 4.5% (258/5,692) of promoter-associated dense-CGIs are methylated in normal PBLs, while 40.5% (129/318) and 40.7% (68/167) of sparse-CGI and non-CGI promoters show such methylation. Methylated promoter CGIs were distributed throughout the genome (Figure S4). Interestingly, most of the identified CGIs were autosomal; in these, the frequency of methylation was 4.0% (223/5,549) for dense-CGI promoters, 39.4% (121/307) for sparse-CGI promoters and 40.9% (65/159) for non-CGI promoters. Except for *MEST*, none of these was associated with known imprinted genes. Together, these data modify the notion that CGI methylation is limited to X chromosome and imprinted genes in normal tissues. Our results also indicate that both non-CGI and sparse-CGI promoters are frequently methylated in normal somatic tissues.

### A Class of Genes with Dense Promoter CGI Methylation and Silencing in Normal Tissues

Since most CGIs previously known to be methylated in normal tissues are located in intragenic regions or promoters of intermediate CpG density, we were surprised to find this exceptional class of autosomal gene promoters associated with dense-CGIs (4.0% of all such CGIs analyzed) that are methylated in normal PBLs. We used bisulfite pyrosequencing to measure DNA methylation quantitatively at such promoter CGIs for 17 genes and confirmed the methylation level of each gene ranged from 68% to 93% in normal PBLs (Table 1). We also measured the methylation of all these genes in two cancer cell lines (the leukemia cell line K562 and the colon cancer cell line RKO) and normal testis. Relative to PBLs, we observed promoter hypomethylation in the cancer cells and testis. This was most striking in K562, where hypomethylation was found in all 17 genes.

**Table 1:**
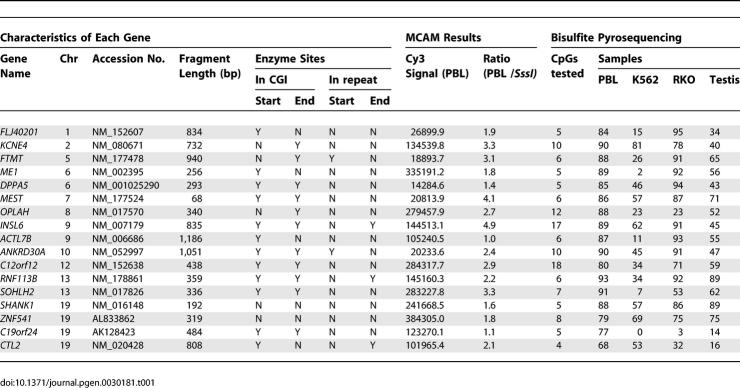
Bisulfite Pyrosequencing Results of Genes Methylated in PBLs by MCAM

**Figure 1:**
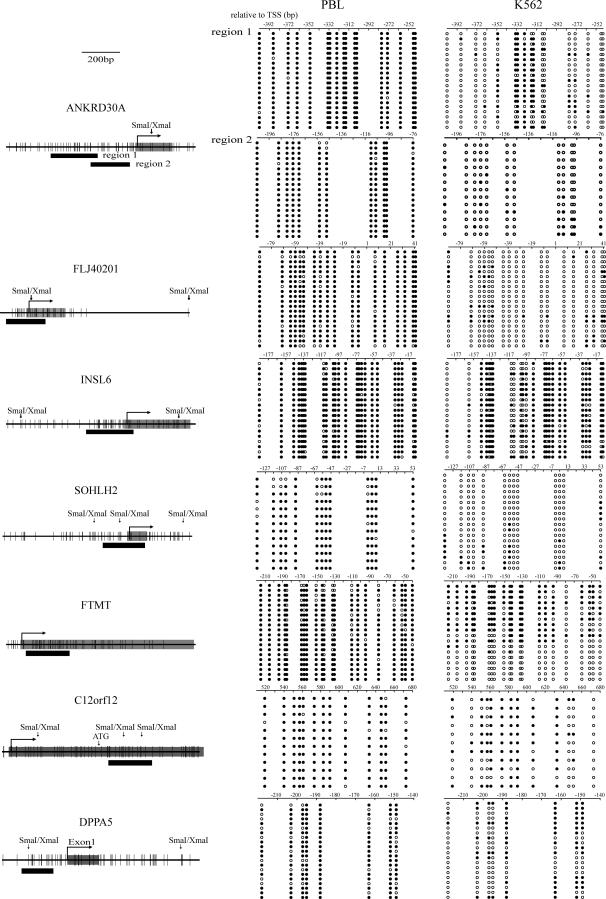
Bisulfite Sequencing of Seven CGI Promoters in PBL and K562 For each gene, a diagram of the CGI promoter is shown on the left. Each **vertical line** represents a single CpG site. The transcription start site, location of exon 1 and restriction enzyme sites are shown on the top. Thick bars indicate the location of regions analyzed by bisulfite-sequencing. Bisulfite sequencing results are shown on the right. Each row represents an individual cloned allele. Circles represent CpG sites and their spacing accurately reflects the CpG density of the region. Black circles, methylated CpG site; white circles, unmethylated CpG site. Dense methylation at the CGI promoters was found in PBLs. In contrast, promoter hypomethylation for all genes was found in K562.

As shown in Table 2, analysis of chromosomal location, CpG density, and GC content of these genes revealed that all have typical CGIs in their promoters, with high CpG densities ranging from 13.2% to 23.2%, and all are located on autosomes with no apparent association with common repetitive sequences or pseudogenes. To determine if methylation affects only a few CpG sites or all CpG sites across the island, we carried out bisulfite cloning and sequencing for seven of these genes, *ANKRD30A*, *FLJ40201*, *INSL6*, *SOHLH2*, *FTMT*, *DPPA5*, and *C12orf12*. All genes were selected on the basis of methylation levels greater than 80% in PBLs by bisulfite pyrosequencing for limited CpG sites. Bisulfite cloning and sequencing provided allele specific methylation data on a larger number of CpG sites, and again showed extensive methylation at all CpG sites within CGIs in normal PBLs (Figure 1, left), but little or no methylation in K562 (Figure 1, right).

**Table 2:**
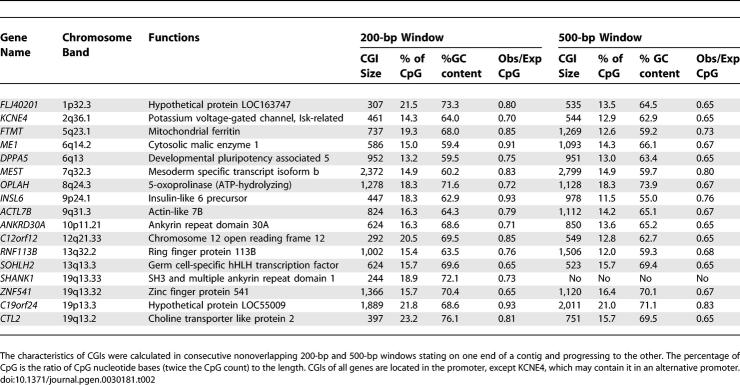
Characteristics of CGI Promoters for Genes Densely Methylated in PBL

**Figure 2:**
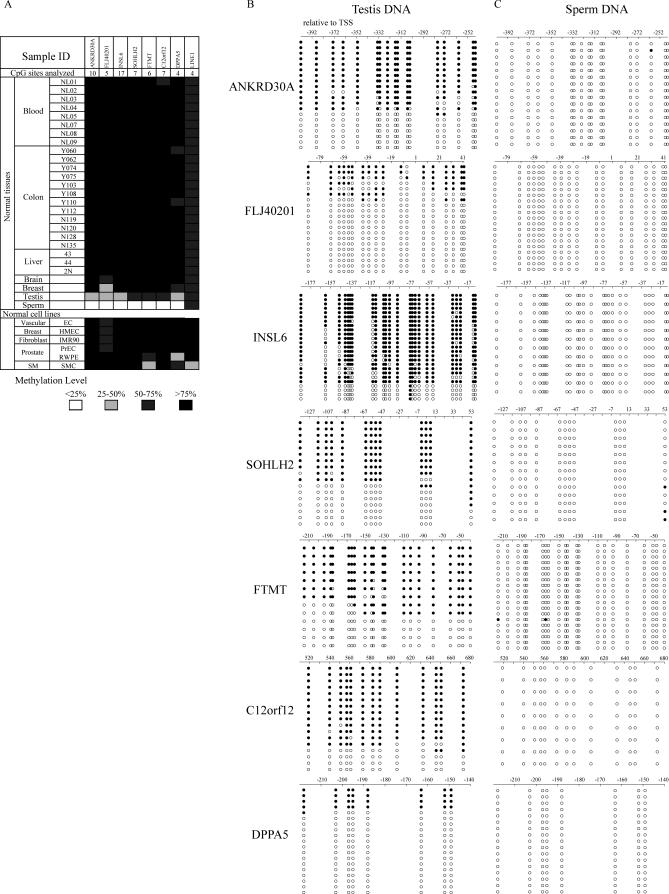
DNA Methylation Analyses in a Panel of Normal Tissues (A) Methylation profiling in normal tissues by bisulfite-pyrosequencing. Promoter methylation of seven identified genes and global methylation by *LINE1* methylation were examined in normal tissues (top) and primary cultured normal cells (bottom). Number of CpG sites analyzed for each gene is indicated in the second row. Methylation level is scored into four scales: Filled black boxes, methylation level greater than 75%; filled dark grey boxes, methylation level from 50% to 75%; filled light grey boxes, methylation level from 25% to 50%, and open boxes, methylation level less than 25%. (B and C) Bisulfite sequencing in sperm (B) and testis (C) DNA identified cells carrying completely unmethylated alleles in all the genes analyzed.

**Figure 3:**
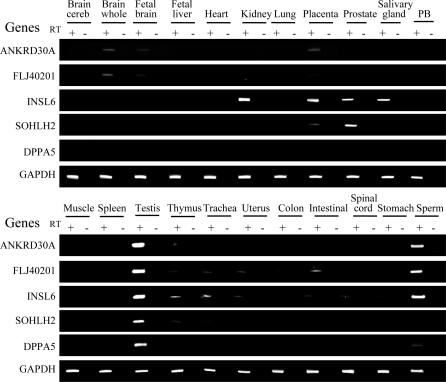
Gene Expression by RT-PCR in a Normal Tissue Panel Tissue types are indicated above the data. The name of each gene amplified is indicated on the left of each column. PCR was performed by using samples prepared with (RT+) or without (RT−) reverse transcriptase. GAPDH was amplified to show the integrity of the RNA.

To assess the extent of tissue and cell-type specific DNA methylation at these CGI promoters, we used quantitative bisulfite pyrosequencing to analyze 33 normal samples derived from ten human tissues: blood, colon, liver, breast, brain, fibroblast, prostate, skeletal muscle, testis and sperm. Dense methylation at these CpG island promoters was found in all tissues except sperm and testis (Figure 2A). Bisulfite cloning and sequencing of sperm DNA identified cells carrying completely unmethylated alleles (Figure 2B). Among the sequences obtained from testis, some alleles were almost completely unmethylated, whereas others were heavily methylated (Figure 2C). As adult testicular tissue contains a mixture of germ line and somatic cells, these results suggest that these unmethylated alleles are derived from germ line cells. We hypothesized that the methylation patterns we observed could predict tissue-specific silencing. To test this, we examined the expression of all genes except two intronless genes (*FTMT* and *C12orf12*) in a cDNA panel from 20 normal tissues. Consistent with the DNA methylation status, all five genes analyzed were strongly expressed in testis (Figure 3). Except *SOHLH2*, expression of four genes (strong expression of three and weak expression of one) was also detected in sperm. However, expression of *SOHLH2* has been reported in oocytes [16]. In contrast, most normal somatic tissues showed no or weak expression of these genes with the exceptions of *INSL6* and *SOHLH2*; *INSL6* was expressed in kidney, placenta, prostate, and salivary gland, and *SOHLH2* was expressed in placenta and prostate. Due to limited tissue availability, we were unable to examine methylation and expression in all tissues; however, we analyzed methylation of *INSL6* and *SOHLH2* in placenta and found promoter hypomethylation for both genes (17.6% for *INSL6* and 20.9% for *SOHLH2*). We conclude that these genes belong to a unique class of promoter CGI associated genes that are methylated and silenced in a tissue-specific manner.

### Gene Ontology and Expression analysis

**Figure 4:**
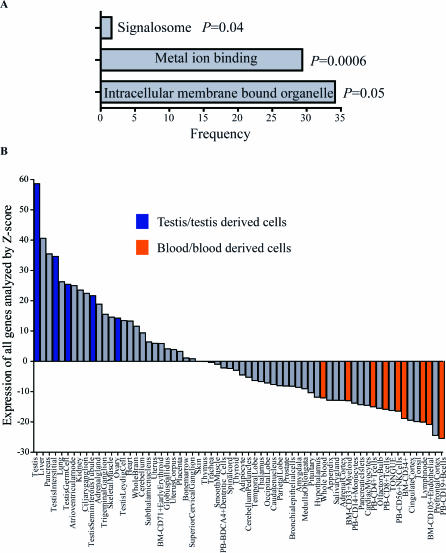
GO Analysis and Tissue-Specific Expression Patterns for Dense-CGI Associated Autosomal Genes Methylated in PBLs (A) GO analysis of this class of genes. Each bar represents the frequency of significant GO terms. (B) Gene expression pattern analysis (GNF database) for 127 genes analyzed by *Z*-score. The y-axis indicates the *Z*-score sum for all genes in a given sample. Each bar represents a normal tissue or cell type. Blue indicates testis or testis-derived cells, orange indicates blood or blood-derived cells, and gray indicates all other tissues/cell types.

To explore potential shared functionality of this class of methylated genes with dense-CGI promoters, we used GOstat gene ontology analysis [17,18] and Benjamini-Hochberg multiple testing correction to identify gene ontology categories that are significantly over-represented. We found most of these genes to be involved in intracellular membrane bound organelle functions (34.1%, *p* = 0.05), followed by metal ion binding (29.4%, *p* = 0.0006) and signalosome functions (1.6%, *p* = 0.04) (Figure 4A). Using published microarray expression databases and applying *Z*-scores to assign equal weight to each gene, we compared the expression levels of 127 genes among 66 different normal tissues and/or cell-types (see Materials and Methods for details). As shown in Figure 4B, expression level analyzed by *Z*-score for all genes was highest in testis, and high in testis-derived cells. Consistent with our methylation data, expression level was greatly decreased in whole blood, as well as various subtypes of blood cells. Indeed, 69% of genes analyzed showed negative *Z*-scores in whole blood relative to other tissue types, which is highly significant (*p* < 2 × 10^−7^, assuming a binomial distribution). These results again suggest that methylation at these promoter CGIs is associated with tissue-specific gene silencing.

### DNA Sequence Analysis

To identify sequence characteristics that could differentiate this class of methylated dense-CGI promoters from the bulk of unmethylated dense-CGI promoters, we first compared CGI size, GC content, and the ratio of observed to expected CpG frequency for these two group of genes identified by MCAM (see Materials and Methods for details). There was no significant difference in CGI length or the ratio of Obs/Exp CpG between the two groups (the average CGI length was 1,157 bp in methylated CGIs versus 1,248 bp in unmethylated CGIs, and the ratio of Obs/Exp CpG was 0.88 in methylated CGIs versus 0.88 in unmethylated CGIs). The methylated CGIs had a slightly higher GC content compared to unmethylated CGIs (66.5 versus 65.6, respectively, *p* = 0.02).

**Figure 5:**
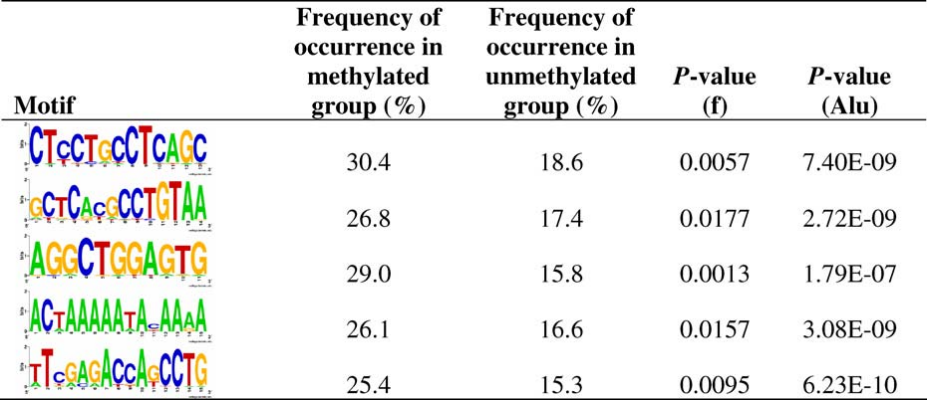
Five Motifs Significantly Enriched in Genes with Methylated CGIs The motifs are represented as sequence logos. The frequency of occurrence was calculated by the number of genes found to match the motif in each group/total number of genes in each group. *p*-Value (f) was calculated from Fisher exact test to test if the motif matches are significantly enriched in the methylated group compared to unmethylated group. *p*-Value (Alu) was calculated by using MAST to match the motif to *Alu* consensus sequence.

Next we used a weight matrix finding algorithm (MEME) [19] and motif alignment and search tool (MAST) [20] to identify sequence motifs that predict methylation patterns. We generated two sets of sequences, one containing 138 sequences (2 kb window) flanking the center of CGIs at methylated genes, and the other containing 2,125 sequences flanking the center of CGIs at unmethylated genes. MEME was used to identify the top 20 significant motifs in each set of sequences (methylated or unmethylated group), and then MAST was used to identify motifs that occur differentially between the methylated and unmethylated groups. Of the top 20 motifs enriched in the methylated group, five showed a significantly higher occurrence in the methylated relative to the unmethylated group (*p* < 0.02 by Fisher exact tests) (Figure 5). In contrast, the top 20 motifs identified in the unmethylated group were present at the same frequency in both groups. Using the TRANSFAC database search, none of these five discriminating motifs was associated with any known transcription factor binding site. Interestingly, however, all were frequently located within sequences homologous to *Alu* sequences (Figure 5).

### Promoter CGI Hypomethylation and Aberrant Expression in Cancer

**Figure 6:**
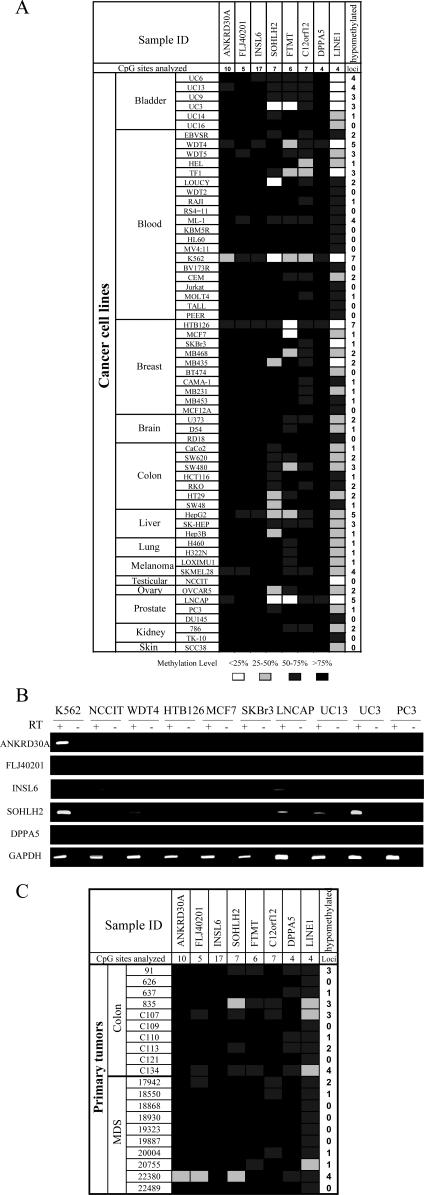
DNA Methylation and Gene Expression Analyses in Cancers (A) Profiles of promoter methylation of seven identified genes and global methylation in cancer cell lines by bisulfite pyrosequencing. (B) Gene expression analysis in selected cancer cell lines. (C) Methylation profiles in primary tumors.

Global genomic hypomethylation and aberrant promoter hypermethylation are epigenetic hallmarks of tumorigenesis [21–23]. We therefore wished to determine if the genes we identified have aberrant promoter methylation in tumors. Methylation analysis of a panel of 61 cancer cell lines from 13 major tumor types including leukemia, melanoma, teratocarcinoma, bladder, breast, brain, ovarian, colon, liver, lung, prostate, kidney, and skin revealed a considerable number of tumors with promoter hypomethylation at the seven promoter CGIs we analyzed. As shown in Figure 6A, the frequency of promoter hypomethylation (defined as methylation level less than 70%) was 9.8% (6/61) for *ANKRD30A*, 9.8% (6/61) for *FLJ40201*, 8.2% (5/61) for *INSL6*, 41.0% (25/61) for *SOHLH2*, 44.2% (27/61) for *FTMT*, 49.2% (30/61) for *C12orf12*, and 6.6% (4/61) for *DPPA5*.

**Figure 7:**
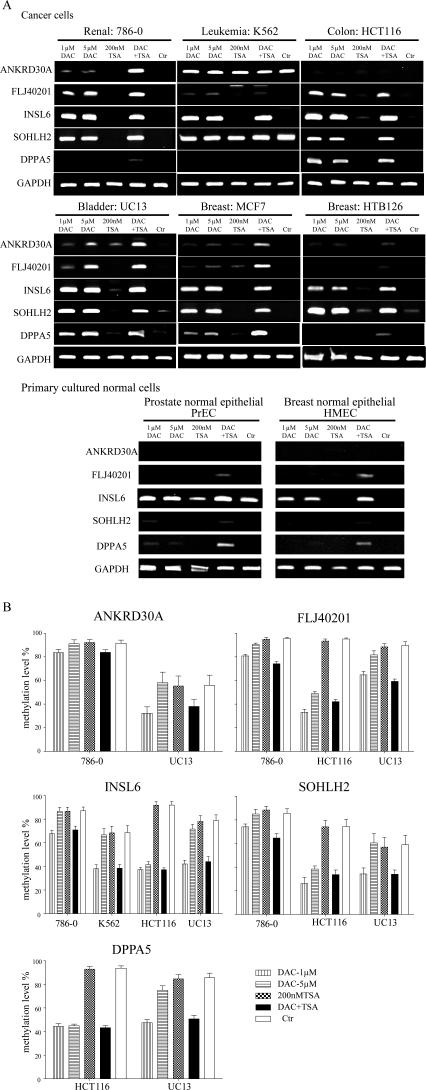
Gene Expression and DNA Methylation Changes after Treatment with DAC or TSA (A) Examples of RT-PCR results. The cell name and tissue type are indicated on the top. The name of each gene amplified is indicated on the left of each column. Cells were treated with 1 μM DAC, 5 μM DAC, 200 nM TSA, a combination of 1 μM DAC and 200 nM TSA (DAC+TSA) or no drug as a control (Ctr). (B) DNA methylation analysis in each promoter region after treatment. Reduced methylation level was detected after DAC and the combination of DAC and TSA treatment. By contrast, TSA did not affect the methylation of any gene.

Next, we examined gene expression in 12 of these cancer cell lines, and observed aberrant expression in association with promoter hypomethylation in several. For example, hypomethylation of *ANKRD30A* corresponded with expression in K562, and relative hypomethylation of *SOHLH2* corresponded with its expression in K562, LNCAP, UC13, and UC3 (Figure 6A and 6B). We also observed a few cases in which hypomethylation does not correlate with gene activation, such as *FLJ40201* in K562. This could occur if, in addition to hypomethylation, gene activation requires specific transcription factors. Among 12 cancer cell lines for which both methylation and expression of these five genes were assessed (Figures 6B and 7A), in only one case (*INSL6* in LNCAP cells) did we observe both aberrant expression and promoter hypermethylation. This expression could possibly originate from a rare subpopulation of hypomethylated cells.

Methylation profiles were also assessed in tumor tissue samples from ten primary colorectal cancer patients and ten myelodysplastic syndrome patients (Figure 6C). As in the cancer cell lines, we observed promoter hypomethylation in the primary tumors; the frequency of hypomethylation was 5% (1/20) for *ANKRD30A*, 20% (4/20) for *FLJ40201*, 0% (0/20) for *INSL6*, 30% (6/20) for *SOHLH2*, 20% (4/20) for *FTMT*, 25% (5/20) for *C12orf12*, and 30% (6/20) for *DPPA5*.

### DNA Methylation Regulates Transcription of This Class of Genes

To investigate the role of DNA methylation in transcriptional regulation of these genes, we examined the effects of treatment with DNA-methyltransferase inhibitor 5-aza-2′-deoxycytidine (DAC) or histone deacetylase inhibitor trichostatin A (TSA) on gene expression in six cancer cell lines and two primary cultures of normal cells (Figure 7A). In most cancer cell lines, reactivation of the silenced genes was observed in response to the treatment with DAC or the combination of DAC and TSA; in contrast, TSA alone had no or little effect. We also observed gene reactivation in the primary cultures of normal cells after DAC and TSA combined. Relatively weak gene reactivation was observed in these normal cells after 1 or 5 μM DAC alone for 3 d; perhaps the result of slower cell division in normal cells, since DAC causes time- and cell division–dependent demethylation by trapping *DNMT1* [24].

Bisulfite pyrosequencing demonstrated that low-dose DAC (1 μM) alone and the combination of DAC with TSA significantly reduced methylation at all gene promoters (Figures 7B and S5), whereas TSA alone did not affect methylation. Interestingly, we observed less hypomethylation in cells treated with high-dose DAC (5 μM), consistent with the notion that low-dose DAC specifically inhibits DNA methylation, whereas high-dose DAC results in cytotoxicity.

**Figure 8:**
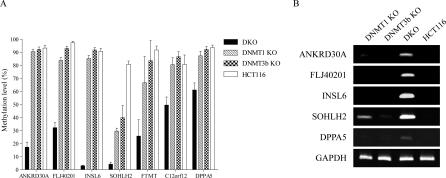
Methylation and Gene Expression Analysis in DNMT Knock-Out Cells (A) DNA methylation analyzed by bisulfite-pyrosequencing in DNA methyltransferase 1 and 3b double knockout (DKO), DNMT1 partial knockout (DNMT1 KO), DNMT3b knockout (DNMT3b KO), and parental HCT116 cells. (B) Gene expression by RT-PCR in HCT116 with and without DNMT knockout.

We next analyzed methylation of each gene in a colorectal cancer cell line (HCT116) after partial knockout of DNMT1, knockout of DNMT3b or knockout of both enzymes (DKO) [25,26]. All genes were heavily methylated in parental cells, and showed dramatic hypomethylation in DKO cells (Figure 8A). For six of the genes (*ANKRD30A*, *FLJ40201*, *INSL6*, *FTMT*, *C12orf12*, and *DPPA5*), slightly reduced methylation was observed in DNMT1 partial knockout cells with almost no changes in DNMT3b knockout cells. By contrast, the methylation level of *SOHLH2* was significantly decreased in DNMT1 knockout cells (from 88% to 26%), and slightly less but still significantly reduced in DNMT3b knockout cells (to 40%). Consistent with methylation results, expression of all genes analyzed was observed in DKO cells, and expression of *SOHLH2* gene was also found in DNMT1 knockout cells (Figure 8B).

## Discussion

During development, a small but significant number of CGIs become methylated and stably silenced [27]. This process of developmentally programmed CGI methylation has been best characterized in genomic imprinting and X chromosome inactivation. Here, using a restriction enzyme–based MCAM approach, we found that non-CGI and sparse-CGI promoters were more susceptible to methylation than dense-CGI promoters, in agreement with a very recent report using an antibody approach to compare methylation profiles between three classes of promoters [28]. Although most dense-CGI promoters remain free of methylation, we found a small exceptional class of such promoters (4.0%) that become methylated in normal somatic tissues and are not associated with X-chromosome or imprinted genes. By detailed characterization of a subset of such genes, we found dense promoter CGI methylation and gene silencing in most normal somatic tissues except germ-line cells. Using RT-PCR and data from published microarray experiments, we confirmed tissue-specific silencing for this class of genes. Further, we showed that inhibition of methylation reactivates expression in these genes. Our results suggest that DNA methylation plays an important role in silencing germ-cell specific genes in somatic tissues. A previous analysis of methylation using RLGS (restriction landmark genome scanning) identified 150 regions (including promoters, exons, and introns) as TDMs (tissue-specific differentially methylated regions) in C57BL/6J mice [29]. By comparing 14 of these mouse TDMs with the human genome, six showed human homologs, and five had conserved tissue-specific methylation and expression, being preferentially expressed in testis [30]. Our results indicate that this pattern affects a relatively large number of genes. On the other hand, other studies failed to identify many dense promoter CpG islands hypermethylated in normal tissues, suggesting that the class of genes we describe here is unique.

It will be important to determine how methylation at these promoter-associated CGIs is established and maintained. One possibility is that methylation is instructed by local sequence features. By comparing DNA sequence flanking the center of CGIs, we identified five sequence motifs significantly enriched in methylated promoter CGIs relative to the bulk of unmethylated CGIs. Although these motifs do not match known transcription factor binding sites, all of them have significant overlap with *Alu,* a family of SINEs (short interspersed elements). *Alu* repeats are rich in CpG dinucleotides and are common targets for DNA methylation. About one-third of methylated CpGs in the genome are located within *Alu* repeats. *Alu* repeats have been proposed as methylation centers for neighboring genes [31] and we hypothesize that *Alu*-related *cis*-regulatory elements may play a role in establishment and/or maintenance of tissue-specific methylation. Experimental approaches such as transfection and transgenic studies will be needed to test this model. Interestingly, while *Alu* repeats are almost completely methylated in most tissues, some, particularly young *Alu* repeats, are almost completely unmethylated in germ line cells [32,33], similar to the genes described here. It remains unclear why tissue-specific gene silencing by DNA methylation is relatively rare, since many genes showing tissue-specific expression are not methylated. What is most exceptional about the genes we describe here is their restricted expression in germ cells. Considering that testis is an immune-privileged site, it is possible that some of these genes, if expressed in somatic tissues, could trigger autoimmune phenomena, justifying the need for a strong mechanism to maintain silencing. In this respect, it is also interesting that we observed hypomethylation of these genes in several cancers. Although the causes and biological effects of cancer-linked hypomethylation remain unclear, such hypomethylation can lead to gene expression that induces an immune response [34,35]. The patterns of methylation and gene expression we observed for this class of genes suggest that some may well be cancer-testis antigens.

In summary, we have identified a group of non-X–linked bona fide promoter CpG islands that are densely methylated in normal somatic tissues, escape methylation in germ line cells, and for which DNA methylation is a primary mechanism of tissue-specific gene silencing.

## Materials and Methods

### Tissue and cell line samples.

Normal tissue samples were obtained from one of the following sources: normal peripheral blood samples from eight healthy donors (three females and five males); 12 normal colon mucosa (five females and seven males), and three normal liver samples (all males) from the MDACC tissue bank; normal brain, breast, placenta, and testis tissues were purchased from BioChain Institute (Hayward, CA); the sperm sample was obtained from a healthy donor and human primary cells were obtained from Cambrex BioScience (East Rutherford, NJ) and American Type Culture Collection (ATCC, Manassas, VA). Tumor samples examined in the present study constitute over 60 cell lines that cover 13 major tumor types (bladder, breast, brain, colon, liver, lung, ovary, prostate, kidney, skin, teratocarcinoma, leukemia, and melanoma) from ATCC. Genomic DNA was extracted using a standard phenol–chloroform method. DNA from the colon cancer cell line HCT116 with DNMT1 knockout, DNMT3b knockout and double knockout (DKO) were kindly provided by Dr. Bert Vogelstein at the Johns Hopkins Kimmel Cancer Center [25,26].

Fully methylated DNA was prepared by treating genomic DNA with M.SssI methylase (New England Biolabs, Beverly, MA). Briefly, 5 μg DNA was incubated at 37 °C in 300 μl containing 20 U of SssI methylase, 320 μM S-adenosylmethionine (SAM, New England Biolabs), and 1× NEB buffer 2 (New England Biolabs). During the incubation, same amounts of SssI methylase and SAM were added one more time to ensure the complete reaction. To verify complete methylation, we performed bisulfite pyrosequencing analysis of seven randomly selected genes that were completely unmethylated before treatment, and found dense methylation at all CpG sites analyzed (41 CpG sties in total) after treatment (Table S2).

### Patient samples.

Primary colon cancer samples from ten colorectal cancer patients and bone marrow samples from ten MDS patients were collected at the Johns Hopkins Hospital and M. D. Anderson Cancer Center in accordance with institutional policies. All patients provided written informed consent. Tumors were selected solely on the basis of availability.

### MCAM.

Methylated CpG island amplification from fully methylated DNA and normal peripheral blood was performed as described [15]. A detailed protocol can be found in the document titled Methylated CpG Island Amplification (in the “Protocols” section; see at http://rd.plos.org/10.1371_journal.pgen.0030181_01). Briefly, 5 μg of genomic DNA was digested with 100 U of methylation-sensitive restriction endonuclease SmaI (New England Biolabs) for 16 h at 20 °C, which cuts unmethylated DNA and leaves blunt ends (CCC/GGG). Subsequently, the DNA was digested with 20 U of methylation-insensitive restriction endonuclease XmaI for 9 h at 37 °C, which leaves sticky ends (C/CCGGG). Adaptors were ligated to the sticky ends. The adaptors were prepared by incubation of the oligonucleotide RMCA12 (5′-CCGGGCAGAAAG-3′) and RMCA24 (5′-CCACCGCCATCCGAGCCTTTCTGC-3′) at 65 °C for 2 min, followed by cooling to room temperature for 30–60 min. 500 ng of digested DNA was ligated to 5 nmol of adaptor using T4 DNA ligase (Invitrogen, Carlsbad, CA). The PCR amplification of sequences flanked by adaptors was performed in a 100 μl reaction mixture comprising 67 mM Tris-HCl (pH 8.8), 4 mM MgCl_2_, 16 mM NH_4_(SO_4_)_2_, 10 mM β-mercaptoethanol, 0.1 mg/ml bovine serum albumin, 5% DMSO, 300 μM dNTP mix, 100 pmol of RMCA24 primer, and 15 units of Taq polymerase (New England Biolabs). The thermocycling conditions were 5 min at 72 °C to fill in the overhanging ends of the ligated DNA fragments, and at 95 °C for 3 min; this was then followed by 25 cycles of 1 min at 95 °C and 3 min at 77 °C, with a final extension of 10 min at 72 °C.

Human promoter arrays were purchased from Agilent Technologies (Agilent, Santa Clara, CA). Microarray protocols, including labeling, hybridization and post-hybridization washing procedures, can be found at http://www.agilent.com/. Briefly, MCA products were labeled with Cy5 (red) for fully methylated DNA or Cy3 (green) for PBLs using a random primed Klenow polymerase reaction (Invitrogen's BioPrime Array CGH Genomic Labeling Kit) at 37 °C for 3 h. Labeled samples were then hybridized to arrays in the presence of human Cot-1 DNA for 40 h at 65 °C. After washing, arrays were scanned on an Agilent scanner and analyzed using Agilent Feature Extraction software at M.D. Anderson Microarray Core Facility.

### Computational hybridization analysis.

We built a database to simulate the performance of MCAM in detecting hypermethylated CpG islands using the SmaI/XmaI isoschizomers. Human genome sequences were downloaded from the UCSC Genome Database (http://genome.ucsc.edu/; version hg17, May 2004). The SmaI/XmaI site “CCCGGG” was searched along each chromosome in a case insensitive fashion. Fragments between two SmaI/XmaI sites were extracted. If the fragment length was between 20 b and 10 kb, the fragment was saved in FASTA format with the first line indicating chromosome number, the starting point of the fragment along chromosome (counting from CCCGGG), and the length of the fragment (including starting and ending CCCGGG).

### Annotation of CGIs and repetitive regions.

CGIs were classified into three classes: dense-CGIs contain a 500 bp area with GC content above 55% and CpG ratio above 0.65; non-CGIs do not contain a 200 bp area with GC content above 50% and CpG ratio above 0.60; and sparse-CGIs are neither dense-CGI nor non-CGI, thus contain CGIs that are either small or have moderate CpG richness. GC content was calculated based on the number of C and G nucleotides within the sequences analyzed. We used the formula cited in Gardiner-Garden et al. [36] to calculate the CpG ratio (Obs/Exp CpG): (Number of CpG × total number of nucleotides in the sequences analyzed) ÷ (number of C × number of G). Repetitive regions were masked in the genome downloaded from UCSC genome database using RepeatMasker/RepBase (versions: RepBase Update 9.11, RM database version 20050112). The databases for SmaI/XmaI fragments were in FASTA format with annotations for (1) chromosome, (2) start point of the fragment along the chromosome, (3) length of the fragment, (4) status of CGI in the starting site, (5) status of CGI in the ending site, (6) if the starting site was within repetitive region, and (7) if the ending site was within repetitive region.

Probe sequences were downloaded from the Agilent website at http://www.agilent.com/. Each probe was BLASTed against all sequences in the SmaI/XmaI database using BLAST v2.2.8 downloaded from NCBI (http://www.genebee.msu.su/blast_new/blastform.php?program=blastn). Probes with multiple BLAT hits were excluded from further study. Probes residing in SmaI/XmaI fragments were identified with the annotation for fragment length, status of CGI, and repetitive sequences.

### Microarray analysis.

We used probes located outside of SmaI/XmaI fragments (length up to 10 kb) for normalization and background calculation. The signal intensity for the probes within the SmaI/XmaI fragments was adjusted for background and analyzed for the ratio between Cy3 and Cy5 signals. All data analysis (sensitivity and reproducibility, correlation between methylation level and chromosome location, CGI, and repetitive sequences) were carried out in Excel (Microsoft). The resulting data sets are accessible in Table S1. We used the following criteria to select hypermethylated probes in PBL (Cy3) relative to fully methylated DNA (Cy5): signal intensity of Cy3 > 3 ×background and ratio of Cy3/Cy5 ≥ 1.5 × background. We performed bisulfite pyrosequencing on 38 randomly selected genes showing higher signal intensity in PBLs, and determined that these criteria most accurately identified hypermethylated loci.

### Cell lines and culture conditions.

Six cancer cell lines from five tumor types: 786–0 (renal), K562 (leukemia), HCT116 (colon), UC13 (bladder), MCF7, and HTB126 (breast) were purchased from ATCC. 786–0, K562, and MCF7 were grown in RPMI 1640 containing 10% fetal bovine serum. HCT116 and HTB126 were grown in high-glucose Dulbecco's modified Eagle's medium containing 10% fetal bovine serum. UC13 was grown in MEM Earle's Salts plus NEAA and 10% fetal bovine serum. Media were purchased from Invitrogen. Two human primary cells, PrEC (prostate) and HMEC (breast), were obtained from Cambrex BioScience and cultured in the media according to the supplier's instructions up to a maximum of five passages. PrECs were grown in prostate epithelial cell growth medium (Clonetics PrEGM bullet kit) containing 0.4% bovine pituitary extract, 5 μg/ml hydrocortisone, 0.5 ng/ml recombinant human epithelial growth factor, 0.5 μg/ml epinephrine, 10 μg/ml transferrin, 5 μg/ml insulin, 0.1 ng/ml retinoic acid, and 6.5 ng/ml triiodothyronine. HMEC cells were grown in mammary epithelial cell basal medium (Clonetics MEMG bullet kit) containing 0.4% bovine pituitary extract, 5 μg/ml hydrocortisone, 0.5 ng/ml recombinant human epithelial growth factor, and 5 μg/ml insulin.

### 5-Aza-2′-deoxycytidine (DAC) and trichostatin A (TSA) treatment of cells.

Cells were split 12–24 h before treatment. Cells were then given one of the following treatments. (1) DAC (1 or 5 μM; Sigma, MO) or phosphate-buffered saline for 72 h. Medium containing DAC or phosphate-buffered saline was changed every 24 h. (2) TSA (200 nM; MP Biomedicals, OH) or an identical volume of ethanol for 24 h. (3) DAC (1 μM) for 48 h followed by DAC (1 μM) and TSA (200 nM) for an additional 24 h. The timing and sequencing of DAC and/or TSA were based on our preliminary studies as well as published studies [37].

### Bisulfite-pyrosequencing for promoter and global DNA methylation analysis.

Bisulfite treatment was performed as reported previously [38]. Briefly, 2 μg of genomic DNA was denatured with 2 M NaOH for 10 min, followed by incubation with 3 M sodium bisulfite (pH 5.0) for 16 h at 50 °C. After treatment, DNA was purified by using a Wizard Miniprep Column (Promega, Madison, WI), precipitated with ethanol, and resuspended in 30 μl of distilled water. 2 μl of the aliquot were used as template for PCR.

We used a quantitative bisulfite pyrosequencing method for all DNA methylation analyses [39,40]. Global DNA methylation was measured by the *LINE1* methylation as previous report [41]. Primer sequences and PCR conditions for bisulfite pyrosequencing assays are summarized in Table S3. The methylation levels at different C sites measured by pyrosequencing were averaged to represent the degree of methylation in each sample for each gene. For each assay, set-up included positive controls (samples after SssI treatment) and negative controls (samples after whole genomic amplification), mixed experiments to rule out bias, and repeated experiments to assess reproducibility. Optimizing annealing temperature of PCR was used to overcome PCR bias as reported [40].

### Bisulfite sequencing.

For selected genes, bisulfite sequencing (performed at the M. D. Anderson Core Sequencing Facility) of cloned PCR products was used to confirm methylation of CpG sites within the CGI promoters. For this analysis, we cloned the PCR products into the TA vector pCR2.1 (Invitrogen) and extracted plasmid DNA from the resulting clones with the use of a QIAprep Spin Miniprep kit (Qiagen, Valencia, CA).

### RNA extraction and RT-PCR.

A panel of RNA from 20 different normal human tissues was obtained from BD Biosciences (multiple tissue cDNA panels) that comprises cerebellum, whole brain, fetal brain, fetal liver, heart, kidney, lung, placenta, prostate, salivary gland, skeletal muscle, spleen, testis, thymus, trachea, uterus, colon, small intestine, spinal cord, and stomach. RNA from normal peripheral blood, sperm and cell lines was prepared by using TRIzol reagents (Invitrogen).

Total RNA (2 μg) was used as a template to generate complementary DNA (cDNA) by random hexamers and M-MuLV reverse transcriptase (Roche, Indianapolis, IN). Reverse-transcription samples without reverse transcriptase also were included as negative controls. One-thirtieth of the cDNA product was used to amplify a 306-bp product of glyceraldehyde-3-phosphate-dehydrogenase (GAPDH) gene as an RNA quality control and one-tenth of the cDNA was used to amplify individual genes. The primer sequences and exons analyzed for RT-PCR were listed in Table S4. PCR conditions were as follows: the reaction volume was 50 μl; initial denaturation was 15 min at 95 °C, followed by 25 cycles (for GAPDH) or 35 cycles (for other genes) of 30 s at 95 °C, 30 s at 55 °C, and 30 s at 72 °C, with a final extension at 72 °C for 10 min. PCR products were visualized on 6% polyacrylamide gels stained with ethidium bromide.

### Bioinformatic analysis.

We used GOstat [18] (http://gostat.wehi.edu.au/) from Gene Ontology Tools (http://www.geneontology.org/GO.tools.shtml) for gene ontology analysis and determined the statistical significance of the overlap with each gene ontology (GO) category using the Fisher exact test. The default multiple testing correction is the Benjamini-Hochberg procedure [42] to control false discovery rate. For gene expression pattern analysis, we downloaded the original profiles from GNF expression database (http://expression.gnf.org/) using probes corresponding to genes identified as dense-CGI promoters methylated in normal blood. From this database, we were able to obtain gene expression data for 127 genes in a panel of 66 normal tissues/cells. To assign equal weight for expression of each gene, we substituted all raw expression values in each data set by their respective *Z*-scores, and the *Z*-score was calculated by (X − μ)/σ, where X stands for expression data of each gene in each sample; μ stands for mean of expression of each gene among all samples; and σ stands for standard deviation. To analyze expression for all genes, each tissue/cell was assigned a score by the sum of *Z*-scores for all genes.

For sequence comparison analyses, we identified two groups of autosomal genes with dense-CGI promoters based on MCAM results: the methylated group, containing 138 genes with signal intensity of PBLs relative to fully methylated DNA greater than 1.5; and the unmethylated group, containing 2,125 genes with signal intensity of PBL relative to fully methylated DNA less than 0.1. The general features of CGIs analyzed in this study include CGI length, GC content, and the ratio of observed to expected CpG frequency. Statistical differences were analyzed by unpaired two-tailed *t* test. Motif analysis was performed as previously reported [43,44]. We generated two sets of sequence databases by extracting 2 kb genomic segments (from the CGI center) for methylated and unmethylated CGIs. Using each dataset as input into the MEME algorithm (http://meme.sdsc.edu/meme/intro.html) [19], we obtained the top 20 “best fit” motifs for both the methylated and unmethylated groups (minLen = 6, maxLen = 50, with a position-specific goodness-of-fit *p*-value less than 10^−6^ after Bonferroni-correction for multiple testing). The 20 top motifs from each group were then individually aligned to the entire two datasets with MAST (http://meme.sdsc.edu/meme/intro.html) [20] to determine the number of occurrences of each motif in methylated and unmethylated promoters. The Fisher exact test was used to compare the frequency of each motif between the two groups. We used TRANSFAC (http://www.gene-regulation.com/cgi-bin/pub/databases/transfac/) to search for matches between motifs with known transcription factor binding site. To evaluate if the overlap between motifs and Alu consensus sequence is significantly greater than expected by chance, we used MAST to search for matches and determine *p*-values.

## Supporting Information

Figure S1 Outline of MCAM Method (A) Schematic diagram of MCAM. A hypothetical fragment of genomic DNA is represented by a solid box, with seven SmaI sites (lollipops). Methylated SmaI sites are indicated by filled lollipops. Fragments A, B, and C are CpG islands with two closely spaced (< 1 kb) SmaI sites. CpG island A is methylated in both samples, while B and C are differentially methylated. Unmethylated SmaI sites are eliminated by digestion with SmaI (which does not cut when its recognition sequence, CCCGGG, contains a methylated CpG); SmaI cleavage leaves blunt ends. The DNA is then digested with the methylation-insensitive SmaI isoschizomer XmaI, which cleaves methylated CCCGGG sites, leaving CCGG overhangs (sticky ends). Adaptors are ligated to these sticky ends, and PCR is performed to amplify the methylated sequences. The amplicons are labeled by Cy3 (green) for sample 1 and Cy5 (red) for sample 2. After hybridization and scanning, hypermethylated fragments in sample 1 result in green signal, hypermethylated fragments in sample 2 result red signal, and equally methylated fragments result in a yellow signal.
(B) Representative results of MCA. 1.5% agarose gel images of MCA amplicons from normal peripheral blood leukocytes (PBL) (sample 1) and fully methylated DNA (sample 2).
(C) Example of microarray scanned image. Differential DNA methylation was compared between fully methylated DNA (Cy5) and normal PBL (Cy3).
(1.6 MB TIF)

Figure S2 Sensitivity and Reproducibility of MCAM (A) Signal intensity of fully methylated DNA (Cy5) for probes located within 10 kb of SmaI/XmaI fragments. Increased signal intensity was found in 87.1% of probes located within 1 kb of the fragments.
(B) Scatter plot analysis of signal intensity (log scale) between fully methylated DNA (y-axis) and normal PBL from a female donor (x-axis) from MCAM. Red indicates probe methylated in fully methylated DNA only and yellow indicates probe methylated in both samples.
(C) Reproducibility of MCAM. Signal intensity of each probe (log scale) from the same sample (PBL) but processed at two different times.
(1.0 MB TIF)

Figure S3 Correlation between MCAM and Bisulfite-Pyrosequencing for 38 Genes All genes showed higher signal intensity in PBLs (A), and genes with dense methylation showed a significantly higher ratio relative to fully methylated DNA (B).
(743 KB TIF)

Figure S4 Chromosomal Distribution of Methylated CGI Promoters Identified from Normal Female Blood Chromosomes number is indicated on the x-axis. Each bar represents the number of genes methylated per chromosome. Black vertical bars indicate gene promoters associated with dense-CGI, gray vertical bars indicate gene promoters associated with sparse-CGI and white vertical bars indicate gene promoters associated with non-CGI.
(875 KB TIF)

Figure S5 INSL6 Promoter Methylation Changes in HCT116 after DAC or TSA Treatment The degree of methylation (y-axis) at 17 single CpGs sites (x-axis) was measured by quantitative bisulfite-pyrosequencing. Reduced methylation was found in cells after DAC or combination of DAC with TSA at all C sites analyzed, in contrast, TSA alone has no effect on methylation.
(703 KB TIF)

Table S1 List of Methylated Genes in PBLs Identified by MCAM (48 KB PDF)

Table S2 Validation of SssI Treatment by Methylation Analysis of 41 CpG Sites (7 KB PDF)

Table S3 Primer Sequences and PCR Conditions for DNA Methylation Analysis (9 KB PDF)

Table S4 Primer Sequences and PCR Conditions for RT-PCR Analysis (7 KB PDF)

### Accession Numbers

The National Center for Biotechnology Information (NCBI) (http://www.ncbi.nlm.nih.gov) accession numbers for the genes studied in this paper are shown in Tables 1 and S1. All microarray datasets in this paper are available at Gene Expression Omnibus (http://www.ncbi.nlm.nih.gov/geo/) under the accession number GSE8810.

## Abbreviations

CGI: CpG island
DAC: 5-aza-2′-deoxycytidine
GO: gene ontology
MAST: motif alignment and search tool
MCA: methylated CpG island amplification
MCAM: methylated CpG island amplification in combination with microarray
MEME: multiple EM for motif mlicitation
PBL: peripheral blood leukocyte
TSA: trichostatin A
