# Supplementary figures and images for: Genome-Wide Profiling of DNA Methylation Reveals a Class of Normally Methylated CpG Island Promoters
Source: PLoS Genet. 2007 Oct 26;3(10):e181. doi: 10.1371/journal.pgen.0030181 (PMC2041996; doi:10.1371/journal.pgen.0030181)

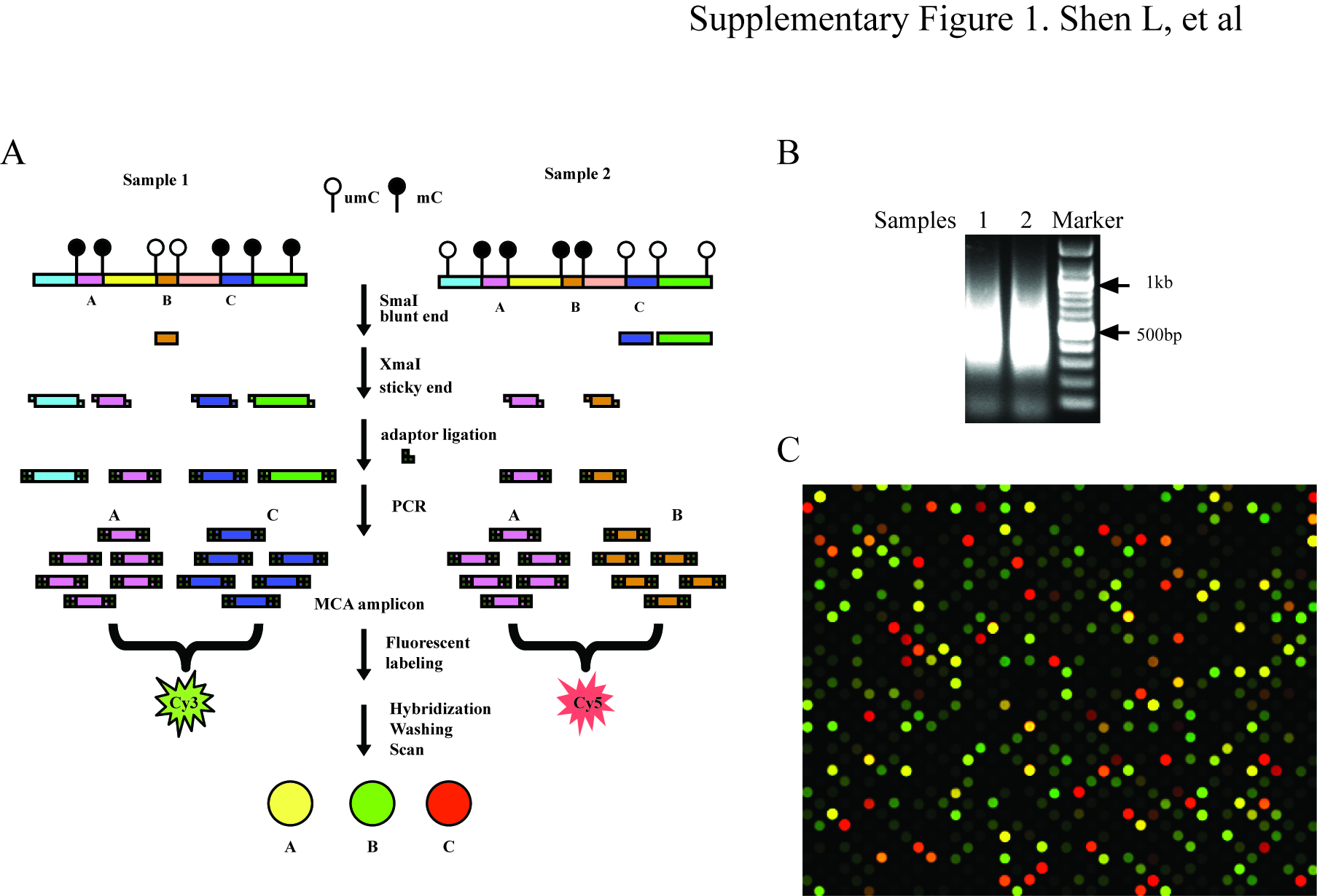

Supplement: Figure S1 — (A) Schematic diagram of MCAM. A hypothetical fragment of genomic DNA is represented by a solid box, with seven SmaI sites (lollipops). Methylated SmaI sites are indicated by filled lollipops. Fragments A, B, and C are CpG islands with two closely spaced (< 1 kb) SmaI sites. CpG island A is methylated in both samples, while B and C are differentially methylated. Unmethylated SmaI sites are eliminated by digestion with SmaI (which does not cut when its recognition sequence, CCCGGG, contains a methylated CpG); SmaI cleavage leaves blunt ends. The DNA is then digested with the methylation-insensitive SmaI isoschizomer XmaI, which cleaves methylated CCCGGG sites, leaving CCGG overhangs (sticky ends). Adaptors are ligated to these sticky ends, and PCR is performed to amplify the methylated sequences. The amplicons are labeled by Cy3 (green) for sample 1 and Cy5 (red) for sample 2. After hybridization and scanning, hypermethylated fragments in sample 1 result in green signal, hypermethylated fragments in sample 2 result red signal, and equally methylated fragments result in a yellow signal. (B) Representative results of MCA. 1.5% agarose gel images of MCA amplicons from normal peripheral blood leukocytes (PBL) (sample 1) and fully methylated DNA (sample 2). (C) Example of microarray scanned image. Differential DNA methylation was compared between fully methylated DNA (Cy5) and normal PBL (Cy3). (1.6 MB TIF) [file pgen.0030181.sg001.tif]

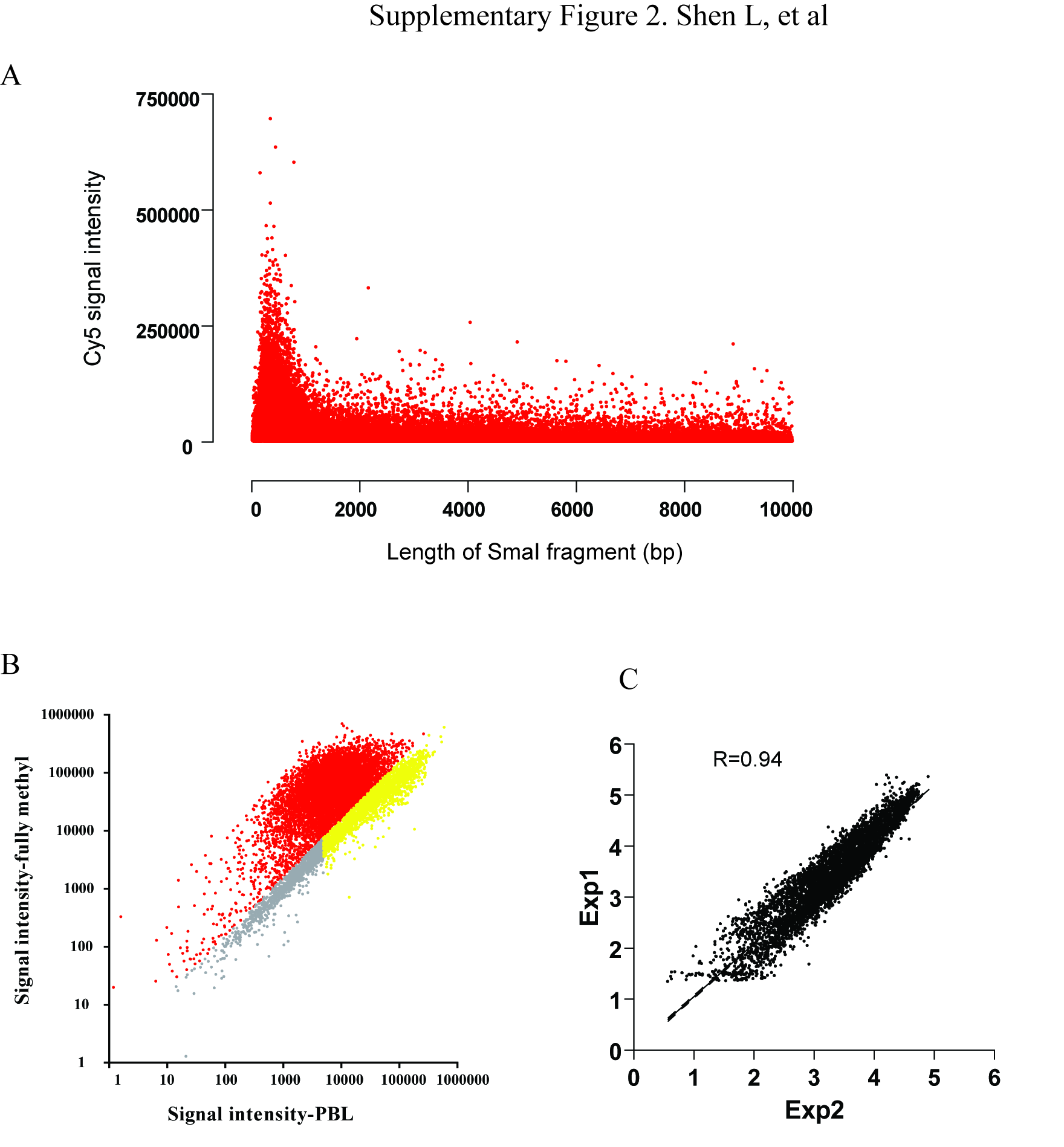

Supplement: Figure S2 — (A) Signal intensity of fully methylated DNA (Cy5) for probes located within 10 kb of SmaI/XmaI fragments. Increased signal intensity was found in 87.1% of probes located within 1 kb of the fragments. (B) Scatter plot analysis of signal intensity (log scale) between fully methylated DNA (y-axis) and normal PBL from a female donor (x-axis) from MCAM. Red indicates probe methylated in fully methylated DNA only and yellow indicates probe methylated in both samples. (C) Reproducibility of MCAM. Signal intensity of each probe (log scale) from the same sample (PBL) but processed at two different times. (1.0 MB TIF) [file pgen.0030181.sg002.tif]

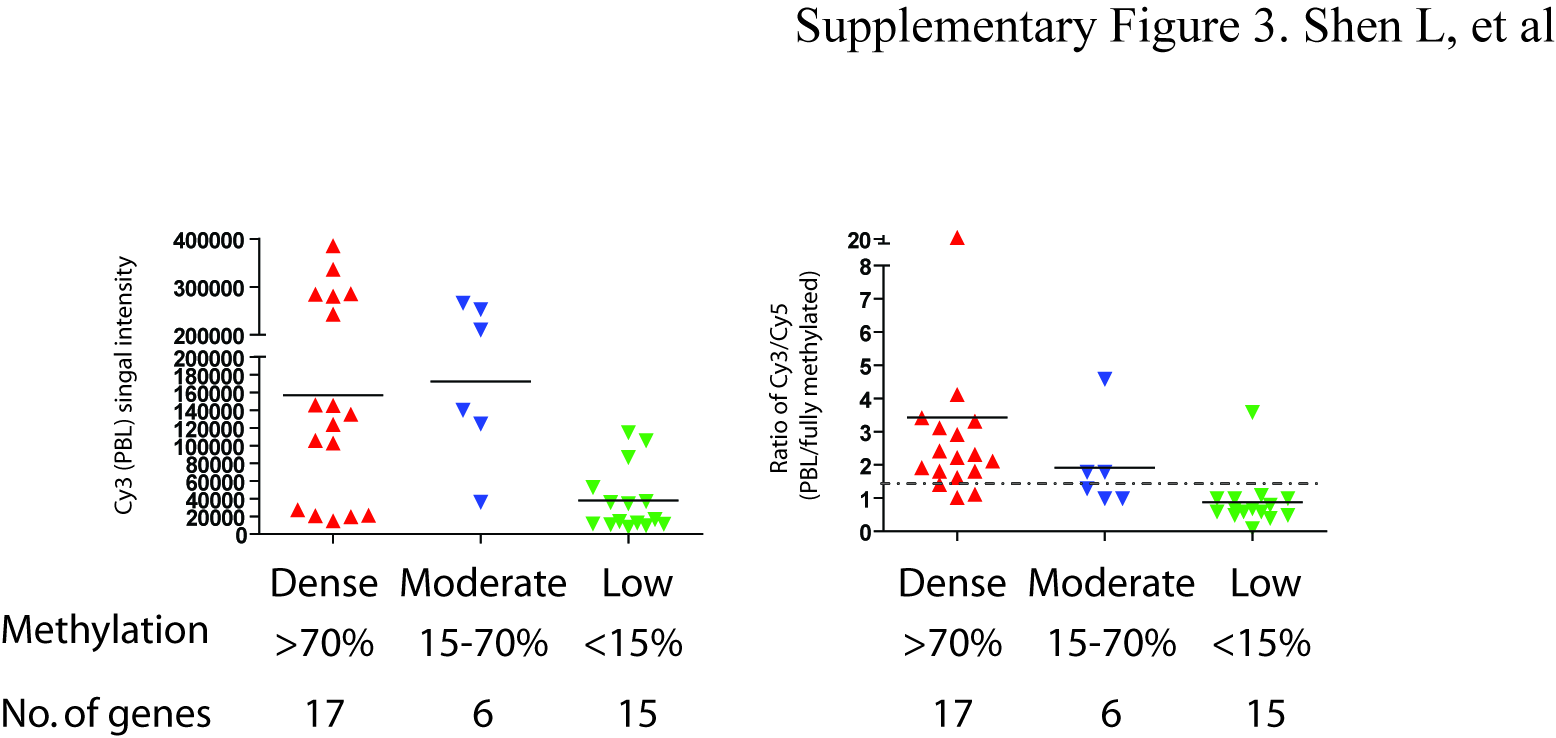

Supplement: Figure S3 — All genes showed higher signal intensity in PBLs (A), and genes with dense methylation showed a significantly higher ratio relative to fully methylated DNA (B). (743 KB TIF) [file pgen.0030181.sg003.tif]

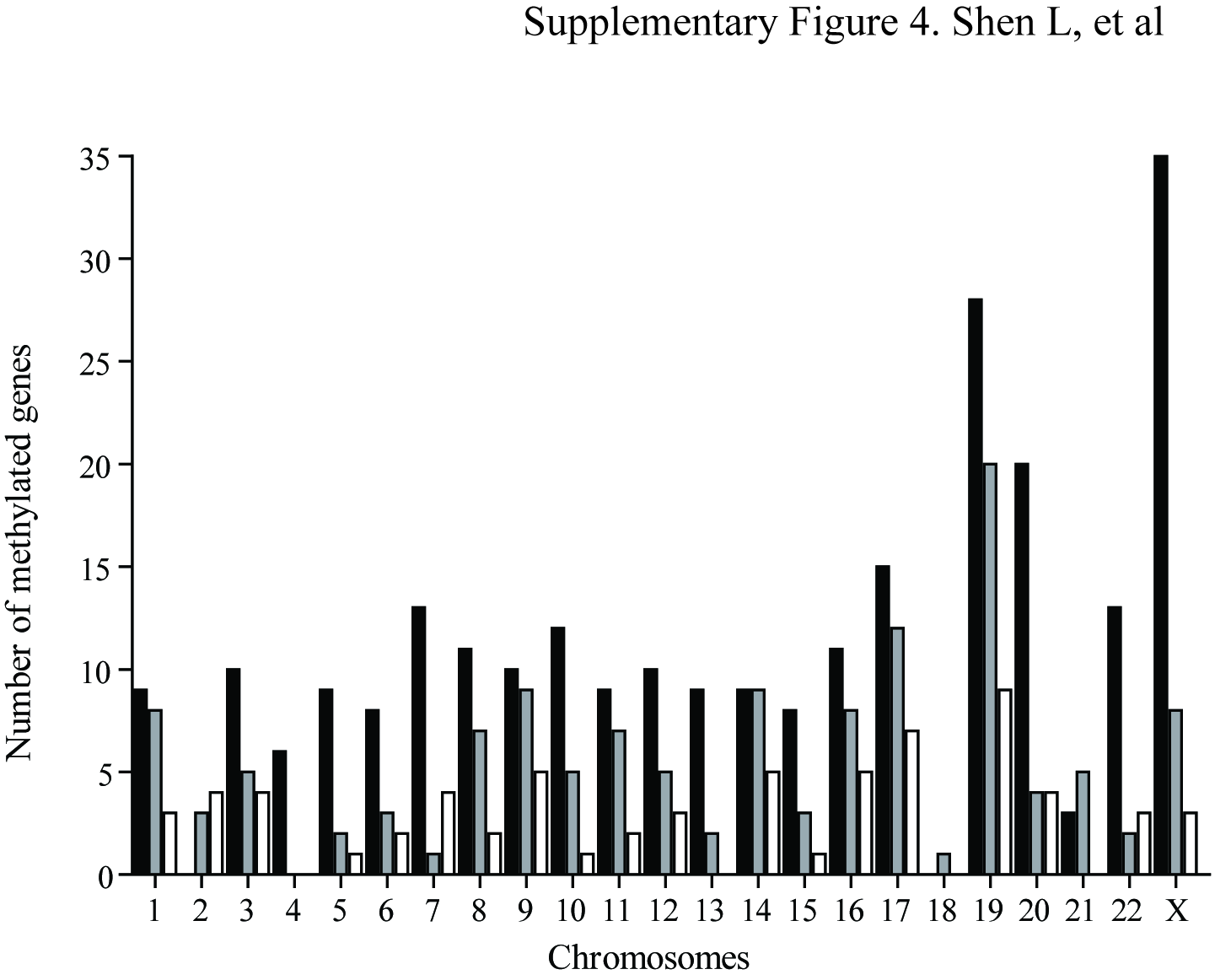

Supplement: Figure S4 — Chromosomes number is indicated on the x-axis. Each bar represents the number of genes methylated per chromosome. Black vertical bars indicate gene promoters associated with dense-CGI, gray vertical bars indicate gene promoters associated with sparse-CGI and white vertical bars indicate gene promoters associated with non-CGI. (875 KB TIF) [file pgen.0030181.sg004.tif]

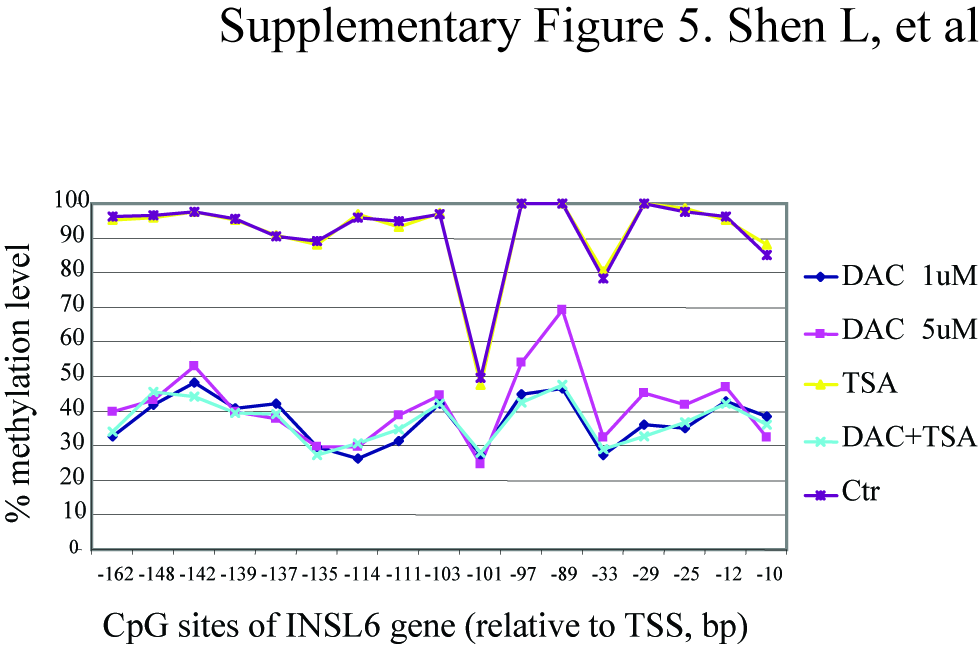

Supplement: Figure S5 — The degree of methylation (y-axis) at 17 single CpGs sites (x-axis) was measured by quantitative bisulfite-pyrosequencing. Reduced methylation was found in cells after DAC or combination of DAC with TSA at all C sites analyzed, in contrast, TSA alone has no effect on methylation. (703 KB TIF) [file pgen.0030181.sg005.tif]
