# Supplementary material for: Genome-Wide Profiling of DNA Methylation Reveals a Class of Normally Methylated CpG Island Promoters
Source: PLoS Genet. 2007 Oct 26;3(10):e181. doi: 10.1371/journal.pgen.0030181 (PMC2041996; doi:10.1371/journal.pgen.0030181)
Supplement: Table S1 — (48 KB PDF) [file pgen.0030181.st001.pdf]

Supplementary table 1. Genes methylated in normal PBL identified by MCAM

Accessions	ProbeName	GeneName	Probe location	CGI status	Length of fragment (bp)	Signal intensity PBL	Signal intensity Fully Methylated	Ratio
NM_003689	A_17_P00075036	AKR7A2	chr1:019383581-019383626	dense-CGI	984	10452.36	6731.86	1.6
NM_033504	A_17_P00124074	TMEM54	chr1:033036997-033037057	dense-CGI	680	141113.40	86066.53	1.6
NM_004983	A_76_P0126357	KCNJ9	chr1:156866801-156866741	dense-CGI	451	19734.75	13601.73	1.5
NM_181644	A_17_P00747943	DKFZp761N1114	chr1:202269905-202269950	dense-CGI	946	7772.31	4430.39	1.8
NM_152666	A_76_P0131246	FLJ40773	chr1:239013571-239013631	dense-CGI	636	7671.34	4372.15	1.8
NM_017629	A_76_P0107188	EIF2C4	chr1:35942233-35942293	dense-CGI	734	11668.28	7907.43	1.5
NM_017629	A_76_P0113819	EIF2C4	chr1:35942573-35942513	dense-CGI	734	10845.13	7022.15	1.5
NM_033067	A_76_P0130086	DMRTB1	chr1:53637013-53637073	dense-CGI	439	16330.39	8934.06	1.8
NM_033067	A_76_P0120255	DMRTB1	chr1:53637327-53637267	dense-CGI	439	45641.77	14552.37	3.1
NM_152607	A_76_P0110290	FLJ40201	chr1:54983818-54983878	dense-CGI	834	26899.86	13808.98	1.9
NM_148974	A_76_P0118935	TNFRSF25	chr1:6460664-6460724	dense-CGI	727	46329.66	18875.07	2.5
NM_005185	A_17_P07043884	CALML3	chr10:005557309-005557354	dense-CGI	711	171667.40	106915.90	1.6
NM_020397	A_17_P07078152	CAMK1D	chr10:012431606-012431651	dense-CGI	719	8392.39	5517.84	1.5
NM_213569	A_17_P07118013	NEBL	chr10:021503345-021503390	dense-CGI	741	15513.33	6391.28	2.4
NM_012071	A_17_P07122048	COMMD3	chr10:022645335-022645380	dense-CGI	573	8749.29	4479.65	2.0
NM_024747	A_76_P0102707	HPS6	chr10:103806097-103806157	dense-CGI	777	8379.04	5282.70	1.6
NM_005308	A_76_P0132627	GRK5	chr10:120956830-120956890	dense-CGI	297	6944.75	4290.36	1.6
NM_198148	A_17_P07543440	CPXM2	chr10:125641023-125641068	dense-CGI	184	128245.60	71545.90	1.8
NM_145235	A_76_P0131620	FANK1	chr10:127574692-127574632	dense-CGI	280	283895.10	83181.28	3.4
NM_145235	A_76_P0118561	FANK1	chr10:127574794-127574854	dense-CGI	427	91900.54	36496.09	2.5
NM_145235	A_76_P0123142	FANK1	chr10:127575020-127574960	dense-CGI	427	267142.90	58532.60	4.6
NM_145235	A_76_P0115092	FANK1	chr10:127575067-127575127	dense-CGI	427	135871.80	64661.97	2.1
NM_145235	A_76_P0116512	FANK1	chr10:127575221-127575161	dense-CGI	427	176919.90	86601.27	2.0
NM_173572	A_17_P07591273	C10orf93	chr10:134606970-134607015	dense-CGI	120	31377.05	18351.36	1.7
NM_052997	A_76_P0116122	ANKRD30A	chr10:37454821-37454881	dense-CGI	1051	20234	8378	2.4
NM_005729	A_76_P0116373	PPIF	chr10:80777447-80777507	dense-CGI	983	7543.16	4870.04	1.5
NM_145869	A_76_P0130924	ANXA11	chr10:81956298-81956238	dense-CGI	707	12048.60	7237.29	1.7
NM_000218	A_17_P07602914	KCNQ1	chr11:002422200-002422245	dense-CGI	711	17951.10	11666.59	1.5
NM_014633	A_76_P0162563	SH2BP1	chr11:10729245-10729185	dense-CGI	694	5561.67	3011.94	1.8
NM_198516	A_76_P0154731	GALNTL4	chr11:11600635-11600575	dense-CGI	982	15871.08	10588.60	1.5
NM_005238	A_17_P08111505	ETS1	chr11:127897129-127897189	dense-CGI	667	12000.29	7149.53	1.7
NM_174902	A_76_P0144849	LOC143458	chr11:35922518-35922578	dense-CGI	658	9185.20	4496.64	2.0
NM_013402	A_76_P0164043	FADS1	chr11:61341379-61341319	dense-CGI	717	10432.34	4573.26	2.3
NM_002032	A_76_P0138536	FTH1	chr11:61491866-61491926	dense-CGI	671	7320.06	4274.11	1.7

NM_022172	A_76_P0149358	PC	chr11:66381380-66381440	dense-CGI	434	35915.96	5004.84	7.2
NM_022172	A_76_P0168886	PC	chr11:66381599-66381539	dense-CGI	434	25164.77	4815.47	5.2
NM_002180	A_76_P0146770	IGHMBP2	chr11:68428192-68428132	dense-CGI	802	12387.38	6573.04	1.9
NM_002180	A_76_P0160801	IGHMBP2	chr11:68428409-68428469	dense-CGI	802	15136.66	7168.29	2.1
NM_024829	A_17_P08205809	FLJ22662	chr12:014612434-014612494	dense-CGI	682	29996.85	15533.65	1.9
NM_138566	A_17_P08373619	GLS2	chr12:055168998-055169058	dense-CGI	637	8759.92	4699.11	1.9
NM_152638	A_17_P08528851	C12orf12	chr12:089850806-089850851	dense-CGI	90	164703.30	46705.02	3.5
NM_152638	A_17_P08528854	C12orf12	chr12:089851148-089851193	dense-CGI	438	284317.70	98058.32	2.9
NM_007076	A_76_P0169197	HYPE	chr12:107411112-107411052	dense-CGI	982	12536.01	7456.58	1.7
NM_152759	A_76_P0143099	MGC35140	chr12:121192977-121192917	dense-CGI	833	21048.40	11975.14	1.8
NM_003428	A_17_P08703726	ZNF84	chr12:132224222-132224267	dense-CGI	384	14209.13	9220.24	1.5
NM_024829	A_76_P0164214	FLJ22662	chr12:14612441-14612501	dense-CGI	682	15417.93	8341.30	1.8
NM_173602	A_76_P0160084	KIAA1463	chr12:49184652-49184712	dense-CGI	999	5293.43	3254.92	1.6
NM_199187	A_76_P0159746	KRT18	chr12:51628458-51628518	dense-CGI	627	8750.81	4159.78	2.1
NM_199187	A_76_P0144369	KRT18	chr12:51628908-51628848	dense-CGI	627	17339.74	8607.13	2.0
NM_018416	A_76_P0152381	FOXJ2	chr12:8075850-8075910	dense-CGI	591	10482.55	4158.62	2.5
NM_020698	A_76_P0170130	TMCC3	chr12:93547055-93546995	dense-CGI	485	5886.93	3333.98	1.8
NM_017826	A_17_P08780520	FLJ20449	chr13:035686476-035686521	dense-CGI	336	283227.80	90167.87	3.1
NM_017826	A_17_P08780521	FLJ20449	chr13:035686608-035686653	dense-CGI	336	277659.30	82892.27	3.3
NM_178861	A_17_P09068707	RNF113B	chr13:097627412-097627457	dense-CGI	359	145160.30	65910.60	2.2
NM_006322	A_76_P0154297	TUBGCP3	chr13:112290572-112290632	dense-CGI	753	4523.53	3009.62	1.5
NM_006322	A_76_P0153182	TUBGCP3	chr13:112290857-112290797	dense-CGI	753	8530.61	4714.42	1.8
NM_024719	A_76_P0163577	GRTP1	chr13:113057389-113057449	dense-CGI	665	40063.51	12487.33	3.2
NM_024719	A_17_P09148213	GRTP1	chr13:113057472-113057532	dense-CGI	665	54645.92	23622.91	2.3
NM_014572	A_76_P0154377	LATS2	chr13:20533769-20533709	dense-CGI	722	11096.36	4955.01	2.2
NM_014572	A_76_P0145455	LATS2	chr13:20534132-20534072	dense-CGI	722	11936.88	6808.95	1.8
NM_016529	A_76_P0157430	ATP8A2	chr13:24940271-24940331	dense-CGI	913	6899.85	3738.15	1.8
NM_017826	A_76_P0138137	FLJ20449	chr13:35686679-35686619	dense-CGI	336	58345.09	32229.54	1.8
NM_025138	A_76_P0144796	C13orf23	chr13:38510129-38510069	dense-CGI	986	19796.09	12180.02	1.6
NM_005845	A_76_P0170084	ABCC4	chr13:94751668-94751728	dense-CGI	371	15180.28	9330.34	1.6
NM_001001715	A_76_P0168210	FARP1	chr13:97627283-97627343	dense-CGI	359	16577.63	10906.35	1.5
NM_001001715	A_76_P0149889	FARP1	chr13:97628419-97628359	dense-CGI	892	39856.88	25827.75	1.5
NM_173159	A_17_P09210464	NPAS3	chr14:032478224-032478275	dense-CGI	245	8792.33	5243.47	1.7
NM_020784	A_17_P09293985	KIAA1344	chr14:052089850-052089895	dense-CGI	675	34725.22	21322.95	1.6
NM_000428	A_17_P09392084	LTBP2	chr14:074148680-074148730	dense-CGI	839	22635.34	12826.74	1.8
NM_006427	A_17_P09532639	SIVA	chr14:104289525-104289570	dense-CGI	362	156208.60	89925.30	1.7
NM_031314	A_76_P0167758	HNRPC	chr14:20807977-20808037	dense-CGI	609	5490.82	3412.85	1.6

NM_002863	A_76_P0144675	PYGL	chr14:50481653-50481593	dense-CGI	932	5277.45	3457.33	1.5
NM_015072	A_76_P0163122	KIAA0998	chr14:75197686-75197626	dense-CGI	870	6098.01	4074.38	1.5
NM_002802	A_76_P0170301	PSMC1	chr14:89792941-89793001	dense-CGI	882	6991.67	4302.91	1.6
NM_006668	A_76_P0137930	CYP46A1	chr14:99220556-99220616	dense-CGI	93	9056.10	5520.57	1.6
NM_007223	A_17_P09604630	GPR	chr15:038000011-038000056	dense-CGI	934	39395.21	25853.55	1.5
NM_182616	A_17_P09822650	MGC61550	chr15:088257492-088257537	dense-CGI	526	134740.70	88569.70	1.5
NM_182616	A_17_P09822652	MGC61550	chr15:088257864-088257920	dense-CGI	526	124906.00	71356.31	1.8
NM_078474	A_76_P0150957	BLP2	chr15:100010193-100010133	dense-CGI	596	4573.47	3049.99	1.5
NM_002487	A_76_P0142717	NDN	chr15:21484094-21484034	dense-CGI	934	6627.05	3565.39	1.9
NM_006293	A_76_P0161092	TYRO3	chr15:39638141-39638201	dense-CGI	487	19852.36	12090.38	1.6
NM_002833	A_76_P0143303	PTPN9	chr15:73659002-73659062	dense-CGI	767	9110.90	5565.12	1.6
NM_002833	A_76_P0160927	PTPN9	chr15:73659335-73659275	dense-CGI	767	6736.34	4550.54	1.5
NM_001004309	A_76_P0165399	MGC75360	chr15:88696820-88696760	dense-CGI	526	18024.57	12345.19	1.5
NM_139057	A_76_P0155590	ADAMTS17	chr15:98699636-98699576	dense-CGI	78	79891.58	47063.05	1.7
NM_003345	A_17_P09885627	UBE2I	chr16:001303985-001304030	dense-CGI	869	148652.70	94503.36	1.6
NM_003345	A_17_P09885628	UBE2I	chr16:001304204-001304249	dense-CGI	869	138268.10	83407.23	1.7
NM_003345	A_17_P09885629	UBE2I	chr16:001304310-001304355	dense-CGI	869	87067.06	50797.81	1.7
NM_032520	A_17_P09885838	GNPTG	chr16:001342115-001342160	dense-CGI	559	31982.06	21491.17	1.5
hsa-mir-193b	A_17_P09939196	hsa-mir-193b	chr16:014304583-014304643	dense-CGI	693	4846.17	3078.26	1.6
NM_001004299	A_17_P09990793	FLJ43980	chr16:045160079-045160124	dense-CGI	855	117657.00	61386.09	1.9
NM_001004299	A_17_P09990795	FLJ43980	chr16:045160429-045160476	dense-CGI	855	78269.37	35733.92	2.2
NM_001004299	A_17_P09990796	FLJ43980	chr16:045160586-045160631	dense-CGI	855	65408.23	29706.27	2.2
NM_001004299	A_17_P09990798	FLJ43980	chr16:045160768-045160813	dense-CGI	855	56969.09	36171.80	1.6
NM_001004299	A_17_P09990799	FLJ43980	chr16:045160893-045160938	dense-CGI	292	91285.31	55555.88	1.6
NM_001039	A_76_P0154863	SCNN1G	chr16:23101213-23101153	dense-CGI	861	12625.44	6530.98	1.9
NM_001039	A_76_P0163901	SCNN1G	chr16:23101331-23101391	dense-CGI	861	10547.67	7178.88	1.5
NM_032815	A_76_P0169233	NFATC2IP	chr16:28864924-28864984	dense-CGI	885	19356.78	5012.99	3.9
NM_020677	A_76_P0140049	HSCARG	chr16:4464619-4464679	dense-CGI	582	22832.07	7172.51	3.2
NM_001004299	A_76_P0162090	FLJ43980	chr16:45160296-45160356	dense-CGI	855	31725.04	12050.92	2.6
NM_001004299	A_76_P0160654	FLJ43980	chr16:45160632-45160572	dense-CGI	855	44045.32	17157.56	2.6
NM_025187	A_76_P0156872	Lin10	chr16:65700790-65700850	dense-CGI	853	18458.00	8123.44	2.3
NM_025187	A_76_P0161518	Lin10	chr16:65701177-65701117	dense-CGI	853	20158.65	5584.08	3.6
NM_025187	A_76_P0143627	Lin10	chr16:65701497-65701557	dense-CGI	853	13065.60	5051.56	2.6
NM_018296mgcs	A_76_P0155496	FLJ11004	chr16:65918326-65918266	dense-CGI	654	19857.70	6749.91	2.9
NM_001740	A_76_P0155551	CALB2	chr16:69949901-69949841	dense-CGI	931	21864.00	10476.20	2.1
NM_145254	A_76_P0168189	LOC124491	chr16:74056023-74056083	dense-CGI	930	9795.08	5230.68	1.9
NM_203411	A_17_P10215307	LOC92162	chr17:007698829-007698878	dense-CGI	503	4564.87	3031.63	1.5

NM_203411	A_17_P10215309	LOC92162	chr17:007699255-007699300	dense-CGI	170	69376.05	44760.83	1.5
NM_152599	A_17_P10219538	FLJ35773	chr17:008642920-008642965	dense-CGI	440	118053.80	68690.36	1.7
NM_152599	A_17_P10219539	FLJ35773	chr17:008643040-008643085	dense-CGI	440	174559.20	107492.40	1.6
NM_031934	A_17_P10273427	RAB34	chr17:024068209-024068254	dense-CGI	111	24423.39	14961.56	1.6
NM_031272	A_17_P10395050	TEX14	chr17:054124078-054124138	dense-CGI	427	69670.05	40302.67	1.7
NM_031272	A_17_P10395052	TEX14	chr17:054124266-054124322	dense-CGI	427	88234.02	41275.73	2.1
NM_001017918	A_17_P10411795	CYB561	chr17:058868637-058868682	dense-CGI	473	55758.38	29936.28	1.9
NM_014595	A_17_P10465226	NT5C	chr17:070640212-070640272	dense-CGI	668	85037.62	49991.68	1.7
NM_006937	A_17_P10465390	SUMO2	chr17:070691484-070691544	dense-CGI	462	160301.30	77418.22	2.1
NM_032134	A_17_P10470266	DKFZP434P0316	chr17:071815801-071815846	dense-CGI	721	58431.81	34914.18	1.7
NM_032134	A_17_P10470267	DKFZP434P0316	chr17:071816087-071816133	dense-CGI	721	113629.80	59418.27	1.9
NM_032134	A_17_P10470269	DKFZP434P0316	chr17:071816298-071816358	dense-CGI	721	80574.10	48196.26	1.7
NM_004127	A_17_P10494939	GPS1	chr17:077603092-077603137	dense-CGI	472	35816.61	20234.92	1.8
NM_006224	A_76_P0144375	PITPNA	chr17:1420467-1420527	dense-CGI	599	45144.23	22922.97	2.0
NM_004140	A_76_P0160626	LLGL1	chr17:18069999-18070059	dense-CGI	670	21629.49	11914.57	1.8
NM_003563	A_76_P0154219	SPOP	chr17:45110678-45110738	dense-CGI	723	6184.16	3039.09	2.0
NM_005450	A_76_P0147203	NOG	chr17:52026365-52026305	dense-CGI	94	11100.78	6654.10	1.7
NM_022036	A_76_P0167498	GPRC5C	chr17:69940620-69940680	dense-CGI	793	10779.34	5231.81	2.1
NM_032134	A_76_P0148110	DKFZP434P0316	chr17:71815816-71815876	dense-CGI	721	51022.84	29580.52	1.7
NM_032134	A_76_P0139591	DKFZP434P0316	chr17:71816122-71816062	dense-CGI	721	55902.70	27887.05	2.0
NM_022158	A_76_P0171811	FN3K	chr17:78285648-78285708	dense-CGI	852	29391.33	18994.02	1.5
NM_152599	A_76_P0170038	FLJ35773	chr17:8643173-8643113	dense-CGI	440	62874.66	33475.15	1.9
NM_152599	A_76_P0166389	FLJ35773	chr17:8643660-8643600	dense-CGI	711	26344.05	17079.33	1.5
NM_004853	A_76_P0139484	STX8	chr17:9419648-9419708	dense-CGI	178	12980.93	6728.30	1.9
NM_017876	A_17_P10851094	RNF126	chr19:000599167-000599212	dense-CGI	639	78944.21	30613.27	2.6
NM_001252	A_17_P10870657	TNFSF7	chr19:006542339-006542386	dense-CGI	726	8069.82	4717.47	1.7
NM_015318	A_17_P10872745	ARHGEF18	chr19:007411085-007411130	dense-CGI	272	219582.90	141765.80	1.5
NM_015318	A_17_P10872749	ARHGEF18	chr19:007411500-007411550	dense-CGI	144	219061.70	84061.47	2.6
NM_198537	A_17_P10909602	FLJ44968	chr19:019500851-019500896	dense-CGI	282	289507.60	174848.20	1.7
NM_016573	A_17_P10910019	GMIP	chr19:019601582-019601642	dense-CGI	970	17653.51	12047.80	1.5
NM_016573	A_17_P10910020	GMIP	chr19:019601708-019601759	dense-CGI	970	15811.43	10510.27	1.5
NM_001864	A_17_P10950298	COX7A1	chr19:041334883-041334928	dense-CGI	46	7069.72	4746.05	1.5
NM_170720	A_17_P10983726	KCNJ14	chr19:053656945-053656990	dense-CGI	635	60768.03	36804.84	1.7
NM_170720	A_17_P10983727	KCNJ14	chr19:053657056-053657101	dense-CGI	635	317601.80	216778.70	1.5
NM_014037	A_17_P10986125	SLC6A16	chr19:054519934-054519979	dense-CGI	247	170378.30	115564.40	1.5
hsa-mir-373	A_17_P10998282	hsa-mir-373	chr19:058983592-058983652	dense-CGI	452	113013.90	74697.61	1.5
hsa-mir-373	A_17_P10998283	hsa-mir-373	chr19:058983759-058983806	dense-CGI	452	102716.20	65290.08	1.6

NM_020428	A_76_P0170540	CTL2	chr19:10597571-10597511	dense-CGI	808	101965.40	49272.56	2.1
NM_001004314	A_76_P0139504	FLJ46419	chr19:12063881-12063941	dense-CGI	236	7171.94	3086.83	2.3
NM_052876	A_76_P0165001	BTBD14B	chr19:13090327-13090387	dense-CGI	197	17911.47	9932.79	1.8
NM_001319	A_76_P0157660	CSNK1G2	chr19:1920345-1920405	dense-CGI	866	66025.28	35496.80	1.9
NM_001319	A_76_P0155656	CSNK1G2	chr19:1920682-1920622	dense-CGI	866	81507.98	53435.89	1.5
NM_001319	A_76_P0166824	CSNK1G2	chr19:1920902-1920962	dense-CGI	866	44639.90	25054.68	1.8
NM_033103	A_76_P0147311	RHPN2	chr19:38248272-38248332	dense-CGI	623	18189.22	4784.20	3.8
NM_153233	A_76_P0144613	FLJ36445	chr19:41191713-41191653	dense-CGI	577	13644.44	6560.95	2.1
NM_032878	A_76_P0144186	MGC15677	chr19:41191916-41191976	dense-CGI	577	8037.21	4300.80	1.9
NM_133466	A_76_P0143168	ZNF545	chr19:41601480-41601540	dense-CGI	897	5014.50	3140.47	1.6
NM_002446	A_76_P0145378	MAP3K10	chr19:45389541-45389481	dense-CGI	940	8053.79	5515.70	1.5
NM_004359	A_76_P0149112	CDC34	chr19:481952-482012	dense-CGI	594	5157.16	3378.79	1.5
NM_006509	A_76_P0168981	RELB	chr19:50196731-50196791	dense-CGI	974	23630.10	16244.86	1.5
NM_012155	A_76_P0151874	EML2	chr19:50834612-50834672	dense-CGI	504	10365.56	4410.51	2.4
NM_012155	A_76_P0157099	EML2	chr19:50835314-50835254	dense-CGI	486	44445.14	19320.79	2.3
NM_002516	A_76_P0152224	NOVA2	chr19:51168979-51168919	dense-CGI	863	18516.01	11219.83	1.7
NM_005184	A_76_P0139577	CALM3	chr19:51796420-51796480	dense-CGI	365	8670.05	5187.56	1.7
NM_012423	A_76_P0162252	RPL13A	chr19:54681531-54681471	dense-CGI	626	36738.30	22319.74	1.6
NM_153375	A_76_P0170572	PLAC2	chr19:5518674-5518734	dense-CGI	388	8046.01	5207.14	1.5
NM_020637	A_76_P0166636	FGF22	chr19:590890-590950	dense-CGI	941	8398.25	5443.99	1.5
NM_020637	A_76_P0138985	FGF22	chr19:591123-591063	dense-CGI	941	8557.38	5339.61	1.6
NM_033113	A_76_P0170136	ZEC	chr19:60685097-60685157	dense-CGI	221	14028.89	7643.43	1.8
NM_002579	A_76_P0148961	PALM	chr19:659213-659273	dense-CGI	539	13787.26	7442.32	1.9
NM_207329	A_17_P01092989	MYADML	chr2:033864726-033864772	dense-CGI	738	125364.60	55217.42	2.3
NM_016441	A_17_P01103651	CRIM1	chr2:036495187-036495232	dense-CGI	58	79174.84	48514.01	1.6
NM_004305	A_17_P01467693	BIN1	chr2:127580687-127580732	dense-CGI	568	21605.75	14399.37	1.5
NM_004807	A_76_P0124402	HS6ST1	chr2:128793064-128793124	dense-CGI	911	11827.34	5584.41	2.1
NM_001024655	A_17_P01480852	LOC554226	chr2:132753535-132753580	dense-CGI	577	246469.00	73784.21	3.3
NM_194317	A_17_P01566389	MGC52057	chr2:150012383-150012428	dense-CGI	288	17920.62	10343.38	1.7
NM_019091	A_76_P0105951	PLEKHA3	chr2:179171172-179171112	dense-CGI	982	10766.51	5932.63	1.8
NM_080671	A_17_P01920363	KCNE4	chr2:223742900-223742960	dense-CGI	732	110220.70	44697.46	2.5
NM_080671	A_17_P01920365	KCNE4	chr2:223743151-223743206	dense-CGI	732	134539.80	41098.08	3.3
NM_004504	A_17_P01941996	HRB	chr2:228162755-228162800	dense-CGI	377	25307.44	14098.72	1.8
NM_014808	A_17_P02006784	FARP2	chr2:242014726-242014771	dense-CGI	369	116882.70	66021.25	1.8
NM_032515	A_17_P02007749	BOK	chr2:242218047-242218092	dense-CGI	486	253472.60	144025.70	1.8
NM_003353	A_76_P0101172	UCN	chr2:27442433-27442373	dense-CGI	487	27445.32	10777.89	2.5
NM_003353	A_76_P0134871	UCN	chr2:27442629-27442689	dense-CGI	487	61029.41	29666.90	2.1

NM_207329	A_76_P0117676	MYADML	chr2:33864812-33864872	dense-CGI	738	124430.60	52355.91	2.4
NM_032107	A_17_P11180349	L3MBTL	chr20:041576626-041576671	dense-CGI	968	35078.53	23621.73	1.5
NM_014835	A_17_P11259590	OSBPL2	chr20:060246794-060246839	dense-CGI	445	24199.12	15729.03	1.5
NM_024299	A_17_P11266131	C20orf149	chr20:061623072-061623117	dense-CGI	879	13937.65	8657.18	1.6
NM_003116	A_76_P0144268	SPAG4	chr20:33667310-33667370	dense-CGI	687	19361.02	10044.21	1.9
NM_032107	A_76_P0160826	L3MBTL	chr20:41576078-41576018	dense-CGI	968	17063.93	9941.64	1.7
NM_032107	A_76_P0168505	L3MBTL	chr20:41576277-41576337	dense-CGI	968	67293.29	46203.88	1.5
NM_032107	A_76_P0162004	L3MBTL	chr20:41576836-41576896	dense-CGI	968	28928.74	19343.53	1.5
NM_002466	A_76_P0164621	MYBL2	chr20:41728886-41728946	dense-CGI	799	12350.58	8315.03	1.5
NM_080606	A_76_P0149679	BHLHB4	chr20:61108843-61108783	dense-CGI	621	8884.56	4815.42	1.8
NM_003224	A_76_P0153222	ARFRP1	chr20:61809555-61809615	dense-CGI	901	24709.55	17010.33	1.5
NM_003224	A_76_P0158612	ARFRP1	chr20:61809620-61809560	dense-CGI	901	19692.90	10832.36	1.8
NM_003681	A_17_P11409035	PDXK	chr21:043962474-043962521	dense-CGI	504	16995.76	10793.71	1.6
NM_001112	A_17_P11414955	ADARB1	chr21:045317417-045317465	dense-CGI	510	156663.20	83717.86	1.9
NM_000100	A_76_P0145062	CSTB	chr21:44021096-44021156	dense-CGI	531	10505.55	4169.24	2.5
NM_000100	A_76_P0149818	CSTB	chr21:44021265-44021205	dense-CGI	531	13696.31	4020.68	3.4
NM_032608	A_17_P11450454	MYO18B	chr22:024462570-024462625	dense-CGI	216	182621.30	66418.46	2.7
NM_182527	A_17_P11467043	CABP7	chr22:028440994-028441039	dense-CGI	349	14159.58	8839.28	1.6
NM_002969	A_17_P11549914	MAPK12	chr22:049003080-049003125	dense-CGI	179	75902.12	45816.61	1.7
NM_014339	A_76_P0162426	IL17R	chr22:15940323-15940263	dense-CGI	560	43478.87	24247.25	1.8
NM_001008496	A_76_P0161352	PIWIL3	chr22:23484197-23484257	dense-CGI	673	38955.32	19973.76	2.0
NM_032608	A_76_P0168908	MYO18B	chr22:24462550-24462610	dense-CGI	216	21314.91	11711.27	1.8
NM_021126	A_76_P0151466	MPST	chr22:35744888-35744828	dense-CGI	339	34787.30	12598.30	2.8
NM_033386	A_76_P0146348	MICAL	chr22:36627211-36627151	dense-CGI	531	7552.16	4397.64	1.7
NM_194326	A_76_P0151744	MGC52010	chr22:38253527-38253587	dense-CGI	953	10386.22	7121.54	1.5
NM_001008572	A_76_P0139032	TTLL1	chr22:41809720-41809660	dense-CGI	815	15237.20	6669.58	2.3
NM_014838	A_76_P0153973	ZBED4	chr22:48567632-48567572	dense-CGI	730	18577.66	5749.82	3.2
NM_018995	A_76_P0151791	MOV10L1	chr22:48831274-48831214	dense-CGI	412	48244.02	27075.39	1.8
NM_138993	A_76_P0154319	MAPK11	chr22:49011425-49011365	dense-CGI	100	11004.14	7389.25	1.5
NM_138433	A_76_P0140056	MGC16635	chr22:49275930-49275870	dense-CGI	282	9461.76	6102.04	1.6
NM_173472	A_17_P02054408	MGC40179	chr3:010124647-010124692	dense-CGI	653	118971.30	29317.79	4.1
NM_182702	A_17_P02215976	TESSP2	chr3:046850368-046850413	dense-CGI	226	78197.55	38925.04	2.0
NM_000316	A_17_P02216261	PTHR1	chr3:046899111-046899162	dense-CGI	825	41447.04	19369.11	2.1
NM_001009996	A_17_P02224984	DALRD3	chr3:049031854-049031912	dense-CGI	201	64482.08	41751.06	1.5
NM_018446	A_17_P02241308	GLT8D1	chr3:052715643-052715694	dense-CGI	772	7271.37	3356.29	2.2
NM_173472	A_76_P0100081	MGC40179	chr3:10124767-10124707	dense-CGI	653	14249.09	7208.04	2.0
NM_000373	A_76_P0101178	UMPS	chr3:125932275-125932335	dense-CGI	937	9079.30	4617.38	2.0

NM_004766	A_76_P0134539	COPB2	chr3:140590982-140591042	dense-CGI	852	7984.72	5450.64	1.5
NM_002628	A_17_P02668733	PFN2	chr3:151171504-151171549	dense-CGI	668	53204.64	14594.30	3.6
NM_012287	A_76_P0132004	CENTB2	chr3:196644737-196644677	dense-CGI	861	13300.60	6910.03	1.9
NM_012157	A_76_P0105655	FBXL2	chr3:33293827-33293767	dense-CGI	939	4826.91	3327.33	1.5
NM_032432	A_17_P02916222	ABLIM2	chr4:008124914-008124959	dense-CGI	340	219284.90	123658.90	1.8
hsa-mir-95	A_17_P02916223	hsa-mir-95	chr4:008125245-008125297	dense-CGI	464	51721.95	31080.77	1.7
hsa-mir-95	A_17_P02916225	hsa-mir-95	chr4:008125505-008125554	dense-CGI	464	123515.40	64661.40	1.9
NM_031279	A_76_P0117546	AGXT2L1	chr4:110041990-110041930	dense-CGI	867	12026.09	5765.77	2.1
NM_004249	A_76_P0128725	RAB28	chr4:13162523-13162583	dense-CGI	508	8540.91	5763.48	1.5
NM_024605	A_76_P0112585	ARHGAP10	chr4:149010266-149010206	dense-CGI	767	7196.41	4514.38	1.6
NM_144979	A_17_P03544796	MGC27016	chr4:156060209-156060254	dense-CGI	452	225043.00	150692.60	1.5
NM_019039	A_17_P03940109	DDX4	chr5:055068598-055068647	dense-CGI	601	134421.40	88274.76	1.5
NM_153610	A_17_P04042255	CMYA5	chr5:079021681-079021731	dense-CGI	491	163017.10	89916.28	1.8
NM_032028	A_17_P04200892	TSSK1	chr5:112798153-112798198	dense-CGI	138	40687.14	21054.41	1.9
NM_177478	A_17_P04238269	FTMT	chr5:121215812-121215860	dense-CGI	141	174155.30	55990.19	3.1
NM_032151	A_76_P0119507	PCBD2	chr5:134268972-134269032	dense-CGI	928	50803.39	27082.04	1.9
NM_004598	A_76_P0129978	SPOCK	chr5:136861855-136861795	dense-CGI	627	53412.11	18124.98	2.9
NM_018834	A_17_P04318150	MATR3	chr5:138656230-138656276	dense-CGI	328	106370.40	57978.40	1.8
NM_004499	A_76_P0134209	HNRPAB	chr5:177563648-177563708	dense-CGI	373	25273.59	7063.58	3.6
NM_017614	A_76_P0123831	BHMT2	chr5:78401341-78401401	dense-CGI	51	6051.11	4045.47	1.5
NM_001012426	A_17_P04688299	FOXP4	chr6:041621678-041621723	dense-CGI	638	5468.15	3709.09	1.5
NM_002395	A_17_P04859572	ME1	chr6:084198057-084198104	dense-CGI	256	335191.20	191092.30	1.8
NM_001431	A_76_P0104868	EPB41L2	chr6:131426107-131426047	dense-CGI	535	4626.74	3135.91	1.5
NM_130900	A_17_P05165916	RAET1L	chr6:150439001-150439046	dense-CGI	433	57741.53	34954.27	1.7
NM_024630	A_76_P0125040	ZDHHC14	chr6:157773258-157773198	dense-CGI	407	14646.22	9777.20	1.5
NM_005453	A_76_P0123302	ZNF297	chr6:33390476-33390416	dense-CGI	741	17551.46	8253.17	2.1
NM_003958	A_76_P0120490	RNF8	chr6:37429678-37429738	dense-CGI	920	10510.45	6279.18	1.7
NM_018665	A_76_P0124694	DDX43-DPPA5	chr6:74159895-74159835	dense-CGI	909	7936	1745	4.5
NM_016587	A_17_P05373127	CBX3	chr7:026014693-026014738	dense-CGI	785	21393.23	12463.07	1.7
NM_006833	A_17_P05646023	COPS6	chr7:099331429-099331474	dense-CGI	69	47818.11	21153.31	2.3
NM_018412	A_17_P05719978	ST7	chr7:116186936-116186983	dense-CGI	500	19462.97	6245.37	3.1
NM_022742	A_17_P05774712	NAG6	chr7:128025167-128025212	dense-CGI	302	27249.62	18516.11	1.5
NM_005631	A_76_P0102533	SMO	chr7:128422693-128422753	dense-CGI	463	9811.80	5949.10	1.6
NM_177525	A_17_P05781778	MEST	chr7:129724727-129724772	dense-CGI	68	20813.86	5020.17	4.1
NM_005397	A_17_P05785928	PODXL	chr7:130698918-130698963	dense-CGI	571	32811.34	18292.87	1.8
NM_022087	A_76_P0123321	GALNT11	chr7:151160271-151160211	dense-CGI	353	13524.00	6945.67	1.9
NM_014766	A_76_P0118905	SCRN1	chr7:29803068-29803128	dense-CGI	641	10982.69	3795.32	2.9

NM_002314	A_76_P0136886	LIMK1	chr7:72942385-72942445	dense-CGI	624	15955.71	10957.67	1.5
NM_177455	A_76_P0118943	MIST1	chr7:97486413-97486353	dense-CGI	41	6311.07	4220.98	1.5
NM_177455	A_76_P0105863	MIST1	chr7:97486498-97486558	dense-CGI	923	35606.72	20671.77	1.7
NM_015395	A_76_P0112748	DKFZP434B0335	chr7:97526455-97526395	dense-CGI	626	6880.96	3532.51	1.9
NM_018842	A_76_P0125537	BAIAP2L1	chr7:97675452-97675392	dense-CGI	654	8913.91	6043.93	1.5
NM_015359	A_17_P06005545	SLC39A14	chr8:022279743-022279788	dense-CGI	986	72557.73	36419.05	2.0
NM_145003	A_76_P0131682	FLJ31164	chr8:143482539-143482479	dense-CGI	929	11515.42	6242.50	1.8
NM_017527	A_76_P0118015	LY6K	chr8:143778743-143778683	dense-CGI	671	16004.79	8102.56	2.0
NM_024736	A_76_P0110685	GSDMDC1	chr8:144709959-144710019	dense-CGI	881	23620.37	10600.59	2.2
NM_032862	A_17_P06533475	TIGD5	chr8:144751686-144751731	dense-CGI	647	11236.14	7633.10	1.5
NM_017570	A_17_P06535205	OPLAH	chr8:145186857-145186902	dense-CGI	340	279457.90	123717.30	2.3
NM_006765	A_76_P0117875	TUSC3	chr8:15441886-15441826	dense-CGI	849	13579.53	7196.82	1.9
NM_032410	A_76_P0126396	HOOK3	chr8:42871383-42871443	dense-CGI	997	6054.45	4118.11	1.5
NM_144651	A_76_P0132286	FLJ25471	chr8:52484570-52484510	dense-CGI	699	37431.05	6274.63	6.0
NM_144651	A_76_P0100174	FLJ25471	chr8:52484741-52484801	dense-CGI	699	104831.30	69985.01	1.5
NM_018444	A_76_P0124120	PPM2C	chr8:94998448-94998508	dense-CGI	714	29980.73	14822.11	2.0
NM_173549	A_76_P0113505	FLJ39553	chr8:99145683-99145623	dense-CGI	861	34124.72	18492.63	1.8
NM_007179	A_17_P06561914	INSL6	chr9:005175453-005175501	dense-CGI	835	139989.30	29961.59	4.7
NM_007179	A_17_P06561917	INSL6	chr9:005175801-005175846	dense-CGI	835	144513.10	42898.87	3.4
NM_007179	A_17_P06561918	INSL6	chr9:005175919-005175973	dense-CGI	835	128884.80	28854.52	4.5
NM_022160	A_17_P06642800	DMRTA1	chr9:022437022-022437067	dense-CGI	464	8883.00	5783.85	1.5
NM_000907	A_17_P06700226	NPR2	chr9:035782065-035782116	dense-CGI	673	198169.40	117760.80	1.7
NM_000907	A_17_P06700229	NPR2	chr9:035782389-035782434	dense-CGI	673	89462.57	52620.41	1.7
NM_002048	A_17_P06796583	GAS1	chr9:086791323-086791375	dense-CGI	548	28423.79	13645.15	2.1
NM_018376	A_76_P0123843	NIPSNAP3B	chr9:104605245-104605305	dense-CGI	872	13933.29	5587.97	2.5
NM_005156	A_17_P06902904	ROD1	chr9:112175026-112175071	dense-CGI	889	15151.89	9983.28	1.5
NM_006752	A_17_P06993719	SURF5	chr9:133244173-133244218	dense-CGI	732	42040.42	25867.00	1.6
NM_017588	A_17_P06997477	WDR5	chr9:134030323-134030368	dense-CGI	82	35760.66	23100.28	1.5
NM_007179	A_76_P0103962	INSL6	chr9:5175577-5175517	dense-CGI	835	64872.32	13241.98	4.9
NM_007179	A_76_P0136785	INSL6	chr9:5175796-5175856	dense-CGI	835	49591.57	13802.31	3.6
NM_007179	A_76_P0108195	INSL6	chr9:5176120-5176060	dense-CGI	835	39722.64	13437.58	3.0
NM_004148	A_76_P0114598	NINJ1	chr9:92975987-92975927	dense-CGI	281	16797.00	11556.13	1.5
NM_032823	A_76_P0119925	C9orf3	chr9:94568062-94568002	dense-CGI	790	4480.14	3013.19	1.5
NM_005333	A_17_P11586728	HCCS	chrX:010889335-010889382	dense-CGI	250	181950.00	103747.60	1.8
NM_153346	A_17_P11620001	CXorf20	chrX:017998232-017998277	dense-CGI	769	194997.10	56117.67	3.5
NM_153346	A_17_P11620002	CXorf20	chrX:017998415-017998460	dense-CGI	769	114288.40	35135.93	3.3
NM_153346	A_17_P11620004	CXorf20	chrX:017998609-017998656	dense-CGI	769	95611.25	31851.72	3.0

NM_153270	A_17_P11634620	RP11-450P7.3	chrX:021435754-021435799	dense-CGI	182	46969.77	31851.41	1.5
NM_153270	A_17_P11634621	RP11-450P7.3	chrX:021436127-021436172	dense-CGI	260	54291.94	20624.25	2.6
NM_153270	A_17_P11634623	RP11-450P7.3	chrX:021436568-021436614	dense-CGI	334	60315.96	31834.98	1.9
NM_153270	A_17_P11634624	RP11-450P7.3	chrX:021436728-021436773	dense-CGI	369	123195.50	59780.41	2.1
NM_153270	A_17_P11634626	RP11-450P7.3	chrX:021436916-021436973	dense-CGI	369	115856.40	47100.68	2.5
NM_173495	A_17_P11642136	PTCHD1	chrX:023111270-023111316	dense-CGI	445	141025.30	75960.10	1.9
NM_173495	A_17_P11642138	PTCHD1	chrX:023111447-023111492	dense-CGI	445	43825.75	29961.71	1.5
NM_002970	A_17_P11643806	SAT	chrX:023560973-023561018	dense-CGI	189	156528.90	99406.97	1.6
NM_002970	A_17_P11643807	SAT	chrX:023561092-023561143	dense-CGI	174	68867.56	41868.27	1.6
NM_152632	A_17_P11696390	CXorf22	chrX:035697756-035697809	dense-CGI	287	73903.70	37934.15	1.9
NM_004683	A_17_P11737537	RGN	chrX:046694321-046694366	dense-CGI	175	71596.93	46112.87	1.6
NM_017602	A_17_P11742373	DKFZp761A052	chrX:048571696-048571741	dense-CGI	397	88167.30	54069.83	1.6
NM_017602	A_17_P11742374	DKFZp761A052	chrX:048571819-048571865	dense-CGI	397	68324.24	46777.89	1.5
NM_007075	A_17_P11742910	WDR45	chrX:048714316-048714361	dense-CGI	913	36722.05	19756.74	1.9
NM_007075	A_17_P11742912	WDR45	chrX:048714683-048714728	dense-CGI	290	28087.62	15410.06	1.8
NM_006150	A_17_P11743134	LMO6	chrX:048799521-048799576	dense-CGI	747	90187.25	46324.38	1.9
NM_006150	A_17_P11743136	LMO6	chrX:048799699-048799746	dense-CGI	747	120300.20	67459.03	1.8
NM_001025449	A_17_P11744696	LOC158572	chrX:049346621-049346666	dense-CGI	960	40667.22	27957.36	1.5
NM_203407	A_17_P11749495	LOC340602	chrX:050982756-050982801	dense-CGI	576	139297.80	52154.28	2.7
NM_203407	A_17_P11749497	LOC340602	chrX:050983015-050983061	dense-CGI	576	274806.30	82405.10	3.3
NM_203407	A_17_P11749498	LOC340602	chrX:050983158-050983203	dense-CGI	576	99350.83	27833.32	3.6
NM_001002838	A_17_P11755566	WNK3	chrX:054267269-054267314	dense-CGI	845	45930.64	29937.08	1.5
NM_020922	A_17_P11755569	WNK3	chrX:054267992-054268042	dense-CGI	865	14844.20	10191.42	1.5
NM_020922	A_17_P11755570	WNK3	chrX:054268145-054268195	dense-CGI	865	18374.37	12625.81	1.5
NM_004429	A_17_P11774923	EFNB1	chrX:067831313-067831359	dense-CGI	816	155109.30	83588.03	1.9
NM_004429	A_17_P11774925	EFNB1	chrX:067831496-067831549	dense-CGI	816	55928.83	33432.95	1.7
NM_004429	A_17_P11774926	EFNB1	chrX:067831611-067831656	dense-CGI	816	84269.76	46153.66	1.8
NM_053281	A_17_P11813509	DACH2	chrX:085209875-085209934	dense-CGI	361	56375.00	30737.22	1.8
NM_212559	A_17_P11850593	XKRX	chrX:099990331-099990376	dense-CGI	329	56058.63	37837.19	1.5
NM_194324	A_17_P11859126	MGC39900	chrX:103023079-103023126	dense-CGI	711	44056.41	26932.69	1.6
NM_194324	A_17_P11859127	MGC39900	chrX:103023196-103023243	dense-CGI	711	59988.23	33995.80	1.8
NM_153448	A_17_P11859903	ESX1L	chrX:103305662-103305707	dense-CGI	798	45373.50	28508.85	1.6
NM_153448	A_17_P11859905	ESX1L	chrX:103306036-103306091	dense-CGI	890	15404.87	10034.13	1.5
NM_153448	A_17_P11859907	ESX1L	chrX:103306612-103306672	dense-CGI	890	8848.93	6079.62	1.5
NM_198465	A_17_P11866458	NRK	chrX:104872794-104872839	dense-CGI	542	98593.53	58264.72	1.7
NM_198465	A_17_P11866459	NRK	chrX:104872906-104872951	dense-CGI	542	121005.40	64494.82	1.9
NM_032227	A_17_P11881771	RP13-360B22.2	chrX:109052520-109052565	dense-CGI	552	124906.30	81455.91	1.5

NM_006667	A_17_P11914205	PGRMC1	chrX:118151584-118151642	dense-CGI	161	69178.87	44920.33	1.5
NM_024528	A_17_P11916452	NKAP	chrX:118859350-118859395	dense-CGI	342	231236.70	149854.30	1.5
NM_024528	A_76_P0154517	NKAP	chrX:118860151-118860091	dense-CGI	718	9791.32	6323.93	1.5
NM_001167	A_17_P11928544	BIRC4	chrX:122719443-122719488	dense-CGI	443	59361.90	39630.63	1.5
NM_003069	A_17_P11947529	SMARCA1	chrX:128382796-128382841	dense-CGI	363	7664.13	4300.37	1.8
NM_139035	A_17_P11947530	SMARCA1	chrX:128382957-128383002	dense-CGI	363	33322.11	16271.34	2.0
NM_001448	A_17_P11964401	GPC4	chrX:132275637-132275682	dense-CGI	415	72814.28	38967.05	1.9
NM_001448	A_17_P11964402	GPC4	chrX:132275750-132275795	dense-CGI	415	81947.92	56123.79	1.5
NM_004484	A_17_P11967400	GPC3	chrX:132844831-132844876	dense-CGI	424	110516.60	40824.79	2.7
NM_003413	A_17_P11980033	ZIC3	chrX:136374293-136374338	dense-CGI	844	41835.09	28554.42	1.5
NM_003413	A_17_P11980034	ZIC3	chrX:136374560-136374605	dense-CGI	844	37210.39	22073.98	1.7
NM_004114	A_17_P11984298	FGF13	chrX:137518530-137518580	dense-CGI	272	66634.87	37032.13	1.8
NM_004114	A_17_P11984300	FGF13	chrX:137518757-137518802	dense-CGI	272	59912.30	31770.69	1.9
NM_017856	A_76_P0155941	FAM51A1	chrX:13807513-13807573	dense-CGI	471	15484.08	10672.94	1.5
NM_005634	A_17_P11991234	SOX3	chrX:139312476-139312521	dense-CGI	135	16533.91	7988.42	2.1
NM_005634	A_17_P11991236	SOX3	chrX:139312707-139312752	dense-CGI	227	83978.38	43632.68	1.9
NM_005634	A_17_P11991238	SOX3	chrX:139313007-139313059	dense-CGI	618	11700.10	6117.59	1.9
NM_005634	A_17_P11991239	SOX3	chrX:139313120-139313167	dense-CGI	618	40520.99	17843.60	2.3
NM_005634	A_17_P11991241	SOX3	chrX:139313334-139313379	dense-CGI	618	34038.19	15148.43	2.2
NM_005634	A_17_P11991244	SOX3	chrX:139313621-139313667	dense-CGI	464	133299.50	87237.11	1.5
NM_000252	A_17_P12023552	MTM1	chrX:149407378-149407423	dense-CGI	737	39592.52	24593.22	1.6
NM_018558	A_17_P12030849	GABRQ	chrX:151477313-151477358	dense-CGI	472	176160.20	109625.30	1.6
NM_000033	A_17_P12034809	ABCD1	chrX:152511420-152511465	dense-CGI	408	102644.50	65139.23	1.6
NM_001018055	A_17_P12039164	CXorf53	chrX:153863722-153863768	dense-CGI	163	23901.27	15767.17	1.5
NM_018196	A_17_P12040125	TMLHE	chrX:154406066-154406111	dense-CGI	326	53751.85	7761.36	6.9
NM_018196	A_17_P12040127	TMLHE	chrX:154406225-154406270	dense-CGI	326	185565.10	10514.26	17.6
NM_173452	A_17_P00104683	FCN3	chr1:027386026-027386071	sparse-CGI	697	18498.68	11253.62	1.6
NM_144622	A_17_P00522833	DCST2	chr1:151818424-151818469	sparse-CGI	539	302859.30	198406.20	1.5
NM_144622	A_17_P00522835	DCST2	chr1:151818614-151818659	sparse-CGI	539	231890.40	123730.00	1.9
NM_032323	A_17_P00527369	MGC13102	chr1:153068264-153068309	sparse-CGI	362	220963.90	133767.40	1.7
NM_014589	A_76_P0116277	PLA2G2E	chr1:19995868-19995808	sparse-CGI	863	43648.04	24346.57	1.8
NM_080431	A_76_P0118068	ARPM2	chr1:2961405-2961465	sparse-CGI	956	76055.43	29812.32	2.6
NM_152374	A_76_P0131685	FLJ38984	chr1:35854594-35854534	sparse-CGI	693	15998.52	4929.80	3.2
NM_022111	A_76_P0110159	CLSPN	chr1:35905830-35905890	sparse-CGI	670	12080.62	4294.45	2.8
NM_005202	A_76_P0104834	COL8A2	chr1:36235244-36235304	sparse-CGI	669	23076.73	11710.40	2.0
NM_005202	A_76_P0109149	COL8A2	chr1:36235582-36235522	sparse-CGI	669	13515.87	7237.44	1.9

NM_001008211	A_17_P07081450	OPTN	chr10:013180617-013180677	sparse-CGI	262	70502.85	44712.84	1.6
NM_001329	A_76_P0123618	CTBP2	chr10:126844452-126844512	sparse-CGI	638	22576.58	6721.62	3.4
NM_194303	A_17_P07587360	C10orf39	chr10:133804972-133805017	sparse-CGI	496	105358.40	48301.07	2.2
NM_194303	A_17_P07587363	C10orf39	chr10:133805276-133805321	sparse-CGI	496	93812.32	50028.10	1.9
nahsa-mi	A_17_P07592624	hsa-mir-202	chr10:134950565-134950610	sparse-CGI	376	242333.10	106790.40	2.3
nahsa-mi	A_17_P07592625	hsa-mir-202	chr10:134950796-134950841	sparse-CGI	376	144709.80	57937.50	2.5
NM_018109	A_76_P0131571	PAPD1	chr10:30678887-30678827	sparse-CGI	735	62473.07	29446.43	2.1
NM_005343	A_17_P07595093	HRAS	chr11:000524057-000524102	sparse-CGI	602	36070.66	16807.79	2.1
NM_001007139	A_17_P07601611	LOC492304	chr11:002107224-002107269	sparse-CGI	74	58449.41	25604.69	2.3
NM_001007139	A_17_P07601613	LOC492304	chr11:002107406-002107456	sparse-CGI	232	196240.10	114026.50	1.7
NM_021801	A_17_P07612347	MMP26	chr11:004965548-004965608	sparse-CGI	460	29850.95	16390.29	1.8
NM_021801	A_17_P07612349	MMP26	chr11:004965708-004965768	sparse-CGI	460	25654.48	17546.35	1.5
NM_024841	A_17_P07753442	FLJ14213	chr11:036433042-036433093	sparse-CGI	957	40966.58	17247.60	2.4
NM_024841	A_17_P07753443	FLJ14213	chr11:036433255-036433302	sparse-CGI	957	66677.24	23971.64	2.8
NM_024841	A_17_P07753445	FLJ14213	chr11:036433466-036433511	sparse-CGI	957	85598.21	34113.80	2.5
NM_024841	A_17_P07753446	FLJ14213	chr11:036433680-036433725	sparse-CGI	957	61319.08	27184.03	2.3
NM_033456	A_17_P07843990	KCNK7	chr11:065120653-065120698	sparse-CGI	476	110517.70	71994.55	1.5
NM_033456	A_17_P07843992	KCNK7	chr11:065120970-065121015	sparse-CGI	476	154463.60	95291.70	1.6
NM_014312	A_76_P0146215	VSIG2	chr11:124127102-124127042	sparse-CGI	475	26983.17	18508.50	1.5
NM_024841	A_76_P0149802	FLJ14213	chr11:36433042-36432982	sparse-CGI	957	19487.71	8862.16	2.2
NM_024841	A_76_P0137800	FLJ14213	chr11:36433260-36433320	sparse-CGI	957	19455.75	7421.20	2.6
NM_024841	A_76_P0138345	FLJ14213	chr11:36433597-36433537	sparse-CGI	957	30377.98	14292.33	2.1
NM_178443	A_76_P0150518	URP2	chr11:63730129-63730189	sparse-CGI	821	53205.22	35039.66	1.5
NM_002527	A_17_P08171436	NTF3	chr12:005473356-005473409	sparse-CGI	511	97150.26	43062.19	2.3
NM_015899	A_17_P08324274	PLEKHA9	chr12:043854567-043854627	sparse-CGI	320	180587.30	91581.15	2.0
NM_002273	A_17_P08357093	KRT8	chr12:051585330-051585382	sparse-CGI	901	24473.58	16598.65	1.5
NM_002273	A_17_P08357096	KRT8	chr12:051585629-051585684	sparse-CGI	901	29404.87	19607.86	1.5
NM_012240	A_17_P08651863	SIRT4	chr12:119201917-119201977	sparse-CGI	703	7689.29	4913.45	1.6
NM_182613	A_17_P08699957	FLJ33915	chr12:131254585-131254634	sparse-CGI	552	161630.30	74577.48	2.2
NM_182613	A_17_P08699959	FLJ33915	chr12:131254739-131254797	sparse-CGI	675	132438.70	66039.44	2.0
NM_182613	A_17_P08699960	FLJ33915	chr12:131254907-131254952	sparse-CGI	675	198302.50	119917.00	1.7
NM_000131	A_17_P09147062	F7	chr13:112807726-112807786	sparse-CGI	843	67643.49	28463.26	2.4
NM_000131	A_17_P09147066	F7	chr13:112808083-112808128	sparse-CGI	843	55936.17	26345.89	2.1
NM_000131	A_17_P09147068	F7	chr13:112808232-112808277	sparse-CGI	843	54031.59	32006.97	1.7
NM_002929	A_76_P0152494	GRK1	chr13:113369631-113369691	sparse-CGI	278	71975.07	49430.66	1.5
NM_002929	A_17_P09149709	GRK1	chr13:113369705-113369750	sparse-CGI	278	271733.10	115366.60	2.4
NM_001805	A_17_P09166421	CEBPE	chr14:022657964-022658009	sparse-CGI	292	261951.50	139979.00	1.9

NM_002471	A_17_P09167585	MYH6	chr14:022927470-022927516	sparse-CGI	716	33475.38	16718.39	2.0
nahsa-mi	A_17_P09167588	hsa-mir-208	chr14:022927864-022927909	sparse-CGI	812	49941.94	31331.10	1.6
nahsa-mi	A_17_P09167589	hsa-mir-208	chr14:022928149-022928204	sparse-CGI	812	58917.83	40613.47	1.5
NM_002471	A_17_P09167695	MYH6	chr14:022946500-022946545	sparse-CGI	639	172790.50	96286.32	1.8
NM_005794	A_17_P09168801	DHRS2	chr14:023177957-023178002	sparse-CGI	327	129422.60	56584.63	2.3
NM_005794	A_17_P09168802	DHRS2	chr14:023178107-023178157	sparse-CGI	327	172336.30	75197.66	2.3
NM_000359	A_17_P09171116	TGM1	chr14:023801205-023801250	sparse-CGI	737	156757.00	49898.11	3.1
NM_000359	A_17_P09171119	TGM1	chr14:023801524-023801569	sparse-CGI	737	157502.50	54298.37	2.9
NM_000359	A_17_P09171121	TGM1	chr14:023801807-023801860	sparse-CGI	737	93349.16	35551.32	2.6
NM_022137	A_17_P09371850	SMOC1	chr14:069414936-069414981	sparse-CGI	373	80892.39	47307.90	1.7
NM_022137	A_17_P09371852	SMOC1	chr14:069415113-069415159	sparse-CGI	373	78313.00	40200.53	1.9
nahsa-mi	A_17_P09517914	hsa-mir-381	chr14:100582022-100582079	sparse-CGI	395	36740.85	14783.61	2.5
nahsa-mi	A_17_P09517917	hsa-mir-382	chr14:100582283-100582328	sparse-CGI	395	245838.60	133682.20	1.8
NM_001031714	A_17_P09532384	C14orf173	chr14:104246845-104246890	sparse-CGI	370	137344.60	84799.34	1.6
NM_001031714	A_17_P09532398	C14orf173	chr14:104249570-104249615	sparse-CGI	627	102476.20	49385.99	2.1
NM_001031714	A_17_P09532401	C14orf173	chr14:104249845-104249890	sparse-CGI	627	86976.09	32213.07	2.7
NM_001031714	A_17_P09532405	C14orf173	chr14:104250317-104250362	sparse-CGI	684	218123.60	71963.25	3.0
NM_001031714	A_17_P09532406	C14orf173	chr14:104250567-104250612	sparse-CGI	684	118895.50	34772.19	3.4
NM_002471	A_76_P0166242	MYH6	chr14:22946362-22946302	sparse-CGI	639	30476.04	20299.67	1.5
NM_002471	A_76_P0151062	MYH6	chr14:22946583-22946643	sparse-CGI	639	14500.77	9991.76	1.5
NM_015492	A_17_P09763526	DKFZP434H132	chr15:073287131-073287176	sparse-CGI	774	111807.30	69342.25	1.6
NM_015492	A_17_P09763528	DKFZP434H132	chr15:073287268-073287315	sparse-CGI	774	104229.80	65089.93	1.6
NM_015492	A_17_P09763530	DKFZP434H132	chr15:073287511-073287556	sparse-CGI	774	213618.10	130570.20	1.6
NM_015492	A_17_P09763531	DKFZP434H132	chr15:073287731-073287780	sparse-CGI	673	146149.20	76321.94	1.9
NM_015492	A_17_P09763532	DKFZP434H132	chr15:073287880-073287925	sparse-CGI	673	98161.77	48791.78	2.0
NM_015492	A_17_P09763533	DKFZP434H132	chr15:073288018-073288063	sparse-CGI	673	124240.90	55020.27	2.3
NM_022767	A_17_P09817042	ISG20L1	chr15:086970497-086970542	sparse-CGI	287	56007.72	30667.96	1.8
NM_022767	A_17_P09817044	ISG20L1	chr15:086970662-086970707	sparse-CGI	287	153705.00	92934.24	1.7
NM_002569	A_17_P09826190	FURIN	chr15:089220004-089220049	sparse-CGI	559	143814.90	75152.54	1.9
NM_002569	A_17_P09826192	FURIN	chr15:089220243-089220288	sparse-CGI	559	409869.90	226855.50	1.8
NM_145294	A_17_P09882889	LOC197336	chr16:000654982-000655042	sparse-CGI	930	32471.22	17362.42	1.9
NM_145294	A_17_P09882891	LOC197336	chr16:000655462-000655507	sparse-CGI	930	58045.54	39837.40	1.5
NM_005823	A_17_P09883299	MSLN	chr16:000750314-000750359	sparse-CGI	163	89752.76	50464.97	1.8
NM_001025190	A_17_P09883405	MPFL	chr16:000773064-000773109	sparse-CGI	255	122625.90	72634.23	1.7
NM_001025190	A_17_P09883406	MPFL	chr16:000773183-000773228	sparse-CGI	255	242108.40	152384.30	1.6
NM_001025190	A_17_P09883407	MPFL	chr16:000773287-000773332	sparse-CGI	884	117245.50	79523.73	1.5
NM_001025190	A_17_P09883409	MPFL	chr16:000773563-000773608	sparse-CGI	884	89506.80	55581.07	1.6

NM_020825	A_17_P09887226	CRAMP1L	chr16:001645802-001645847	sparse-CGI	419	297404.00	184839.20	1.6
NM_020825	A_17_P09887227	CRAMP1L	chr16:001645932-001645977	sparse-CGI	419	230068.20	132107.30	1.7
NM_020825	A_17_P09887228	CRAMP1L	chr16:001646192-001646237	sparse-CGI	113	66081.00	18216.91	3.6
NM_020825	A_17_P09887229	CRAMP1L	chr16:001646313-001646358	sparse-CGI	121	43707.38	15748.24	2.8
NM_020825	A_17_P09887230	CRAMP1L	chr16:001646421-001646466	sparse-CGI	257	125458.00	79429.24	1.6
NM_020825	A_17_P09887231	CRAMP1L	chr16:001646548-001646593	sparse-CGI	257	26717.10	16282.39	1.6
NM_032271	A_17_P09889373	TRAF7	chr16:002153427-002153472	sparse-CGI	672	158142.40	77559.94	2.0
NM_022452	A_17_P09984069	FBS1	chr16:030583394-030583439	sparse-CGI	330	187114.70	112069.40	1.7
NM_022452	A_17_P09984071	FBS1	chr16:030583535-030583580	sparse-CGI	330	250921.30	138546.60	1.8
NM_022452	A_17_P09984073	FBS1	chr16:030583831-030583885	sparse-CGI	343	176820.30	106964.30	1.7
NM_022452	A_17_P09984074	FBS1	chr16:030583957-030584002	sparse-CGI	343	336368.20	188684.20	1.8
NM_014699	A_17_P09985485	ZNF646	chr16:030995388-030995433	sparse-CGI	739	54340.14	35917.56	1.5
NM_014699	A_17_P09985487	ZNF646	chr16:030995553-030995606	sparse-CGI	739	78030.35	49235.91	1.6
NM_014699	A_17_P09985488	ZNF646	chr16:030995760-030995813	sparse-CGI	739	81582.89	48418.29	1.7
NM_014298	A_76_P0166285	QPRT	chr16:29596462-29596402	sparse-CGI	741	7372.19	5036.71	1.5
NM_005823	A_76_P0138418	MSLN	chr16:750170-750230	sparse-CGI	163	19390.94	12816.68	1.5
NM_001976	A_17_P10203549	ENO3	chr17:004796096-004796141	sparse-CGI	365	143121.00	55391.94	2.6
NM_017986	A_17_P10204000	GPR172B	chr17:004878384-004878429	sparse-CGI	724	128830.00	45389.75	2.8
NM_017986	A_17_P10204002	GPR172B	chr17:004878680-004878732	sparse-CGI	724	55650.78	18188.87	3.1
NM_002055	A_17_P10336411	GFAP	chr17:040348395-040348440	sparse-CGI	215	184335.80	126171.20	1.5
NM_000727	A_17_P10427244	CACNG1	chr17:062470215-062470275	sparse-CGI	464	66406.24	38101.08	1.7
NM_000727	A_17_P10427248	CACNG1	chr17:062470863-062470908	sparse-CGI	652	155033.70	85000.37	1.8
NM_000727	A_17_P10427249	CACNG1	chr17:062471244-062471289	sparse-CGI	652	181952.60	84674.13	2.1
NM_000727	A_17_P10427251	CACNG1	chr17:062471394-062471439	sparse-CGI	652	131920.50	77556.81	1.7
NM_019020	A_17_P10486304	TBC1D16	chr17:075539846-075539891	sparse-CGI	801	71289.43	45418.82	1.6
NM_015229	A_76_P0164446	KIAA0664	chr17:2564825-2564885	sparse-CGI	441	27104.90	14273.80	1.9
NM_080704	A_76_P0156888	TRPV1	chr17:3448578-3448518	sparse-CGI	772	23513.83	13171.17	1.8
NM_021939	A_76_P0166520	FKBP10	chr17:37226714-37226654	sparse-CGI	773	42995.16	14257.92	3.0
NM_021939	A_76_P0162713	FKBP10	chr17:37226935-37226995	sparse-CGI	773	56023.77	17123.33	3.3
NM_002512	A_76_P0165481	NME2	chr17:46595625-46595685	sparse-CGI	703	20296.55	11311.60	1.8
NM_000727	A_76_P0145633	CACNG1	chr17:62470612-62470552	sparse-CGI	464	38070.88	25791.50	1.5
NM_000727	A_76_P0170646	CACNG1	chr17:62470826-62470886	sparse-CGI	652	50670.02	29714.19	1.7
NM_000727	A_76_P0170363	CACNG1	chr17:62471152-62471092	sparse-CGI	652	73839.62	39422.95	1.9
NM_000727	A_76_P0147714	CACNG1	chr17:62471372-62471432	sparse-CGI	652	59067.38	30524.33	1.9
NM_080913	A_76_P0161521	ASGR2	chr17:6960155-6960215	sparse-CGI	887	18612.96	6212.42	3.0
NM_144679	A_76_P0169759	FLJ31528	chr17:76835880-76835820	sparse-CGI	752	23232.84	8064.56	2.9
NM_024619	A_76_P0147485	FN3KRP	chr17:78266817-78266877	sparse-CGI	606	13005.41	4124.69	3.2

NM_002385	A_17_P10833217	MBP	chr18:072858273-072858325	sparse-CGI	465	253573.30	63273.49	4.0
NM_002385	A_17_P10833218	MBP	chr18:072858422-072858467	sparse-CGI	465	192895.80	57385.50	3.4
NM_002385	A_76_P0138649	MBP	chr18:72858199-72858139	sparse-CGI	465	55486.15	34467.91	1.6
NM_002385	A_76_P0168991	MBP	chr18:72858419-72858479	sparse-CGI	465	47065.64	31690.51	1.5
NM_173481	A_17_P10851373	C19orf21	chr19:000701854-000701899	sparse-CGI	626	148338.70	81558.77	1.8
NM_173481	A_17_P10851374	C19orf21	chr19:000701972-000702017	sparse-CGI	626	293962.20	158084.50	1.9
NM_032447	A_17_P10875200	FBN3	chr19:008118152-008118197	sparse-CGI	360	154158.70	105800.80	1.5
NM_020230	A_17_P10880350	PPAN	chr19:010082461-010082512	sparse-CGI	840	66474.46	43175.96	1.5
NM_015329	A_17_P10908721	KIAA0892	chr19:019316769-019316824	sparse-CGI	278	131326.60	82354.85	1.6
NM_015329	A_17_P10908723	KIAA0892	chr19:019316932-019316977	sparse-CGI	278	231922.30	139090.40	1.7
NM_005031	A_17_P10946742	FXYD1	chr19:040321839-040321884	sparse-CGI	773	108167.30	56838.41	1.9
NM_005031	A_17_P10946743	FXYD1	chr19:040321951-040321996	sparse-CGI	773	95149.09	53190.97	1.8
NM_005031	A_17_P10946744	FXYD1	chr19:040322096-040322142	sparse-CGI	773	49300.84	24049.94	2.0
NM_005031	A_17_P10946745	FXYD1	chr19:040322308-040322355	sparse-CGI	773	51047.10	27767.37	1.8
NM_033557	A_17_P10955826	YIF1B	chr19:043487830-043487875	sparse-CGI	777	79172.64	30623.88	2.6
NM_033557	A_17_P10955827	YIF1B	chr19:043488008-043488053	sparse-CGI	777	130026.70	57671.61	2.3
NM_032255	A_17_P10981353	ZNF541	chr19:052739624-052739669	sparse-CGI	319	384305.00	217941.40	1.8
NM_016148	A_17_P10990436	SHANK1	chr19:055911925-055911970	sparse-CGI	192	241668.50	153345.80	1.6
nahsa-mi	A_17_P10993885	hsa-mir-99b	chr19:056887151-056887196	sparse-CGI	957	140232.50	83293.32	1.7
NM_020230	A_76_P0161655	PPAN	chr19:10082596-10082656	sparse-CGI	840	63272.87	35361.97	1.8
NM_020230	A_76_P0149633	PPAN	chr19:10082937-10082877	sparse-CGI	922	9881.81	6610.27	1.5
NM_002566	A_76_P0160011	P2RY11	chr19:10083174-10083234	sparse-CGI	922	11158.09	6662.58	1.7
NM_002566	A_76_P0140436	P2RY11	chr19:10083468-10083408	sparse-CGI	922	26591.03	18018.76	1.5
NM_145045	A_76_P0139614	MGC20983	chr19:11402562-11402502	sparse-CGI	974	29684.40	12293.18	2.4
NM_145045	A_76_P0159745	MGC20983	chr19:11402722-11402782	sparse-CGI	974	21546.15	7856.59	2.7
NM_014173	A_76_P0158141	HSPC142	chr19:17240598-17240538	sparse-CGI	921	12987.69	5811.66	2.2
NM_138442	A_76_P0168653	LOC115098	chr19:17901605-17901545	sparse-CGI	627	23448.62	7185.54	3.3
NM_002248	A_76_P0142429	KCNN1	chr19:17945999-17945939	sparse-CGI	315	21585.64	14328.91	1.5
NM_005755	A_76_P0167462	EBI3	chr19:4175793-4175733	sparse-CGI	624	45448.82	10266.17	4.4
NM_005755	A_76_P0139852	EBI3	chr19:4175850-4175910	sparse-CGI	624	58925.14	13960.73	4.2
NM_005755	A_76_P0171853	EBI3	chr19:4177085-4177025	sparse-CGI	989	23046.21	9207.02	2.5
NM_024907	A_76_P0146922	FBXO17	chr19:44158922-44158862	sparse-CGI	853	16280.70	3420.29	4.8
NM_002774	A_76_P0152931	KLK6	chr19:56165470-56165530	sparse-CGI	836	14304.87	5295.41	2.7
NM_214710	A_76_P0140910	PRSSL1	chr19:646865-646925	sparse-CGI	979	8525.71	4763.05	1.8
NM_173481	A_76_P0151431	C19orf21	chr19:702402-702342	sparse-CGI	547	61776.09	35985.97	1.7
NM_002777	A_76_P0166357	PRTN3	chr19:787874-787814	sparse-CGI	885	13527.73	6097.23	2.2
NM_001004699	A_76_P0144073	OR2Z1	chr19:8701621-8701561	sparse-CGI	627	22845.00	10200.84	2.2

nahsa-mi	A_17_P01897024	hsa-mir-26b	chr2:219092887-219092937	sparse-CGI	500	80170.23	27651.29	2.9
NM_007127	A_76_P0131672	VIL1	chr2:219114241-219114181	sparse-CGI	921	143350.70	56843.03	2.5
NM_022134	A_76_P0119509	GAL3ST2	chr2:242436126-242436186	sparse-CGI	813	24867.87	16808.47	1.5
NM_004118	A_17_P11131771	FKHL18	chr20:029896841-029896886	sparse-CGI	349	206796.80	124046.50	1.7
NM_017454	A_17_P11201303	STAU	chr20:047239193-047239253	sparse-CGI	417	49635.84	32055.69	1.5
NM_178463	A_17_P11261092	C20orf166	chr20:060554500-060554545	sparse-CGI	741	87194.35	50480.90	1.7
NM_178463	A_17_P11261093	C20orf166	chr20:060554829-060554874	sparse-CGI	741	127589.60	86024.93	1.5
NM_152302	A_17_P11262759	C20orf158	chr20:060982210-060982255	sparse-CGI	497	187026.70	109548.10	1.7
NM_152302	A_17_P11262763	C20orf158	chr20:060982594-060982639	sparse-CGI	258	133081.30	70260.35	1.9
NM_198078	A_17_P11401414	C21orf121	chr21:042315307-042315355	sparse-CGI	770	54663.41	36645.57	1.5
NM_000394	A_17_P11406742	CRYAA	chr21:043462122-043462169	sparse-CGI	810	38129.26	19803.71	1.9
NM_000394	A_17_P11406744	CRYAA	chr21:043462341-043462386	sparse-CGI	810	88456.52	43569.80	2.0
NM_206965	A_17_P11419755	FTCD	chr21:046399597-046399642	sparse-CGI	866	156730.80	100103.80	1.6
NM_198078	A_76_P0154234	C21orf121	chr21:42315368-42315308	sparse-CGI	770	55080.23	26625.68	2.1
NM_198078	A_76_P0159293	C21orf121	chr21:42315519-42315579	sparse-CGI	770	35258.58	17881.64	2.0
NM_194309	A_76_P0155335	C21orf125	chr21:43694024-43694084	sparse-CGI	897	54237.84	35347.60	1.5
NM_173510	A_76_P0166146	FLJ33814	chr22:27491893-27491953	sparse-CGI	986	13457.72	3375.04	4.0
NM_153609	A_76_P0143157	TMPRSS6	chr22:35823799-35823859	sparse-CGI	718	46183.60	19696.46	2.3
NM_002673	A_17_P02222378	PLXNB1	chr3:048440760-048440805	sparse-CGI	976	54487.82	31354.17	1.7
NM_001008269	A_17_P02223388	LOC440955	chr3:048634359-048634404	sparse-CGI	638	49767.46	24569.35	2.0
NM_007354	A_17_P02575176	GR6	chr3:129775946-129776006	sparse-CGI	336	169653.00	88917.73	1.9
NM_000174	A_17_P02576773	GP9	chr3:130263520-130263565	sparse-CGI	234	256980.30	98645.30	2.6
NM_139209	A_17_P02631006	GRK7	chr3:142980075-142980120	sparse-CGI	236	238309.80	142238.80	1.7
NM_022147	A_76_P0109614	IFRG28	chr3:188568713-188568653	sparse-CGI	975	8767.08	5147.50	1.7
NM_022147	A_76_P0119753	IFRG28	chr3:188568932-188568992	sparse-CGI	975	7336.84	4119.78	1.8
NM_022304	A_76_P0118603	HRH2	chr5:175042509-175042569	sparse-CGI	957	23461.53	12408.63	1.9
NM_022304	A_17_P04481998	HRH2	chr5:175042608-175042668	sparse-CGI	957	7116.48	4833.93	1.5
NM_022304	A_17_P04482002	HRH2	chr5:175042991-175043037	sparse-CGI	957	55865.70	37693.50	1.5
NM_001031799	A_17_P04496677	RASGEF1C	chr5:179496755-179496801	sparse-CGI	600	201820.90	107221.60	1.9
NM_018950	A_76_P0124503	HLA	chr6:29798665-29798725	sparse-CGI	666	10744.63	3104.45	3.5
NM_002754	A_76_P0120508	MAPK13	chr6:36204659-36204599	sparse-CGI	961	27751.13	8885.33	3.1
NM_001760	A_76_P0114954	CCND3	chr6:42024722-42024782	sparse-CGI	709	23814.86	6658.04	3.6
NM_017781	A_17_P05260811	CYP2W1	chr7:000796070-000796115	sparse-CGI	746	545277.30	335725.40	1.6
NM_017781	A_17_P05260812	CYP2W1	chr7:000796226-000796271	sparse-CGI	92	14180.46	4233.02	3.3
NM_017781	A_17_P05260813	CYP2W1	chr7:000796436-000796481	sparse-CGI	558	71732.40	44602.46	1.6
NM_017781	A_76_P0112037	CYP2W1	chr7:795672-795612	sparse-CGI	746	68839.49	29200.88	2.4
NM_017781	A_76_P0123551	CYP2W1	chr7:795918-795978	sparse-CGI	746	90855.03	38611.56	2.4

NM_017781	A_76_P0107603	CYP2W1	chr7:796462-796522	sparse-CGI	558	78830.77	51823.11	1.5
NM_033512	A_17_P06321832	TSPYL5	chr8:098360078-098360130	sparse-CGI	634	89391.77	49341.48	1.8
NM_033512	A_17_P06321833	TSPYL5	chr8:098360236-098360293	sparse-CGI	634	54092.72	30118.32	1.8
NM_178857	A_76_P0108235	RP1L1	chr8:10566933-10566993	sparse-CGI	899	44357.72	28836.12	1.5
NM_178857	A_76_P0131303	RP1L1	chr8:10567195-10567135	sparse-CGI	611	92283.83	59467.28	1.6
NM_178857	A_76_P0127127	RP1L1	chr8:10567379-10567439	sparse-CGI	611	64864.51	39493.62	1.6
NM_002066	A_17_P06530833	GML	chr8:143912981-143913026	sparse-CGI	967	41528.31	19855.68	2.1
NM_002066	A_76_P0135776	GML	chr8:143913050-143913110	sparse-CGI	967	19461.73	11018.35	1.8
NM_002066	A_76_P0106226	GML	chr8:143913361-143913301	sparse-CGI	967	28659.37	10865.59	2.6
NM_002066	A_76_P0108341	GML	chr8:143913581-143913641	sparse-CGI	967	23038.31	12341.01	1.9
NM_198488	A_17_P06533885	FLJ46072	chr8:144883353-144883398	sparse-CGI	251	142831.70	91567.53	1.6
NM_201379	A_17_P06534808	PLEC1	chr8:145100727-145100772	sparse-CGI	447	328586.00	194666.10	1.7
NM_017767	A_17_P06536092	SLC39A4	chr8:145612601-145612646	sparse-CGI	879	191783.90	111538.00	1.7
NM_017767	A_17_P06536093	SLC39A4	chr8:145612819-145612864	sparse-CGI	879	136890.90	94084.42	1.5
NM_017767	A_17_P06536094	SLC39A4	chr8:145612978-145613023	sparse-CGI	879	161590.50	106179.40	1.5
NM_130849	A_17_P06536095	SLC39A4	chr8:145613159-145613204	sparse-CGI	255	278005.20	176792.30	1.6
NM_013432	A_76_P0104105	NFKBIL2	chr8:145632284-145632224	sparse-CGI	622	171901.60	67956.92	2.5
NM_013432	A_76_P0125551	NFKBIL2	chr8:145632505-145632565	sparse-CGI	622	136458.70	66970.46	2.0
NM_033512	A_76_P0130327	TSPYL5	chr8:98360040-98360100	sparse-CGI	634	32502.56	15475.24	2.1
NM_080655	A_76_P0121584	MGC17337	chr9:100266920-100266860	sparse-CGI	645	15659.90	3871.83	4.0
NM_080655	A_76_P0119922	MGC17337	chr9:100267140-100267200	sparse-CGI	645	14550.66	3781.18	3.8
NM_203305	A_17_P06971430	C9orf132	chr9:127792251-127792299	sparse-CGI	817	44044.75	29124.06	1.5
NM_203305	A_76_P0123284	EEIG1	chr9:127794375-127794315	sparse-CGI	571	17871.01	5090.79	3.5
NM_001013652	A_17_P06972087	LOC389791	chr9:127971170-127971215	sparse-CGI	251	163413.10	66849.98	2.4
NM_002957	A_76_P0109937	RXRA	chr9:134519034-134518974	sparse-CGI	797	40300.63	25020.38	1.6
NM_002957	A_17_P06998812	RXRA	chr9:134519247-134519292	sparse-CGI	797	207960.20	121250.90	1.7
NM_002957	A_76_P0102558	RXRA	chr9:134519255-134519315	sparse-CGI	797	105568.90	53711.25	2.0
NM_002957	A_17_P06998814	RXRA	chr9:134519384-134519429	sparse-CGI	797	266752.10	137082.20	1.9
NM_002957	A_17_P06999040	RXRA	chr9:134555947-134555992	sparse-CGI	957	107583.10	57625.62	1.9
NM_002957	A_17_P06999042	RXRA	chr9:134556104-134556149	sparse-CGI	957	68785.93	39859.59	1.7
NM_001012415	A_76_P0119562	C9orf157	chr9:135817221-135817161	sparse-CGI	557	76108.52	4431.50	17.2
NM_001012415	A_76_P0133879	C9orf157	chr9:135817440-135817500	sparse-CGI	136	22443.88	11935.98	1.9
NM_001012415	A_76_P0116575	C9orf157	chr9:135817779-135817719	sparse-CGI	792	143038.60	68706.64	2.1
NM_001012415	A_76_P0130533	C9orf157	chr9:135817963-135818023	sparse-CGI	792	77610.74	40224.89	1.9
NM_203347	A_17_P07010195	UNQ2541	chr9:136935252-136935297	sparse-CGI	927	65338.01	40506.54	1.6
NM_203347	A_17_P07010196	UNQ2541	chr9:136935451-136935499	sparse-CGI	927	91140.12	52458.37	1.7
NM_001606	A_76_P0135415	ABCA2	chr9:137193230-137193290	sparse-CGI	995	9179.89	4688.06	2.0

NM_001606	A_76_P0126606	ABCA2	chr9:137193569-137193509	sparse-CGI	995	20426.85	7372.86	2.8
NM_001606	A_76_P0116168	ABCA2	chr9:137193715-137193775	sparse-CGI	995	11395.65	5102.68	2.2
NM_000832	A_17_P07011733	GRIN1	chr9:137307716-137307761	sparse-CGI	662	196057.30	111264.80	1.8
NM_205849	A_17_P11576285	FAM9B	chrX:008811102-008811147	sparse-CGI	380	143101.70	40551.93	3.5
NM_174901	A_17_P11596564	FAM9C	chrX:012822539-012822584	sparse-CGI	282	165130.70	66742.09	2.5
NM_130776	A_17_P11751377	XAGE3	chrX:052779864-052779909	sparse-CGI	987	77435.94	20902.85	3.7
NM_145799	A_17_P11915792	6-Sep	chrX:118610146-118610191	sparse-CGI	341	162314.90	96657.31	1.7
NM_173493	A_76_P0160780	PASD1	chrX:150402385-150402445	sparse-CGI	713	30335.49	11307.22	2.7
NM_173493	A_76_P0137878	PASD1	chrX:150402669-150402609	sparse-CGI	713	93805.17	39532.15	2.4
NM_173493	A_17_P12027082	PASD1	chrX:150402730-150402777	sparse-CGI	713	108848.40	31618.23	3.4
NM_001011543	A_17_P12029434	MAGEA10	chrX:150977429-150977476	sparse-CGI	536	184215.10	81012.47	2.3
NM_000054	A_17_P12035550	AVPR2	chrX:152691316-152691361	sparse-CGI	707	115530.70	58740.88	2.0
NM_000054	A_17_P12035552	AVPR2	chrX:152691510-152691555	sparse-CGI	707	91864.43	53439.21	1.7
NM_172377	A_17_P12037837	CTAG2	chrX:153445187-153445235	sparse-CGI	810	66070.59	19781.84	3.3
NM_172377	A_76_P0155989	CTAG2	chrX:153445252-153445312	sparse-CGI	810	16232.44	8812.08	1.8
NM_020997	A_17_P00835626	LEFTY1	chr1:222383807-222383860	non-CGI	591	213762.70	129396.00	1.7
NM_198074	A_17_P00929390	OR2C3	chr1:244021534-244021586	non-CGI	829	18343.54	12215.84	1.5
NM_014466	A_76_P0117289	TEKT2	chr1:36216174-36216114	non-CGI	556	10698.42	6321.64	1.7
NM_032807	A_17_P07045601	FBXO18	chr10:005976048-005976095	non-CGI	387	12235.19	4958.40	2.5
NM_002457	A_17_P07597377	MUC2	chr11:001063938-001063983	non-CGI	724	122051.40	83361.12	1.5
NM_002457	A_17_P07597384	MUC2	chr11:001064957-001065002	non-CGI	245	71632.75	38817.61	1.8
NM_006278	A_17_P08100477	ST3GAL4	chr11:125781641-125781686	non-CGI	724	62738.20	42342.93	1.5
NM_006278	A_17_P08100479	ST3GAL4	chr11:125781772-125781817	non-CGI	724	133812.20	78169.30	1.7
NM_004963	A_17_P08206334	GUCY2C	chr12:014740322-014740382	non-CGI	512	111769.30	28162.50	4.0
NM_170754	A_17_P08357622	TENC1	chr12:051730476-051730522	non-CGI	788	260830.50	177945.20	1.5
NM_004963	A_76_P0148090	GUCY2C	chr12:14740507-14740567	non-CGI	512	30442.55	11337.29	2.7
NM_198232	A_17_P09156133	RNASE1	chr14:020340194-020340245	non-CGI	885	38343.78	25051.50	1.5
NM_198232	A_17_P09156135	RNASE1	chr14:020340403-020340463	non-CGI	885	22716.16	15558.98	1.5
NM_198232	A_17_P09156138	RNASE1	chr14:020340677-020340725	non-CGI	885	33999.27	21882.55	1.6
NM_198235	A_17_P09156142	RNASE1	chr14:020341032-020341082	non-CGI	454	60629.86	37894.07	1.6
NM_004050	A_17_P09167177	BCL2L2	chr14:022846548-022846608	non-CGI	415	15344.93	8944.60	1.7
NM_004050	A_17_P09167179	BCL2L2	chr14:022846738-022846795	non-CGI	415	23461.23	13355.41	1.8
NM_015299	A_17_P09172083	KIAA0323	chr14:023967373-023967418	non-CGI	785	71407.78	22362.09	3.2
NM_015299	A_17_P09172086	KIAA0323	chr14:023967665-023967723	non-CGI	785	129785.10	39591.21	3.3
NM_001002236	A_17_P09484734	SERPINA1	chr14:093926976-093927023	non-CGI	449	75640.12	30049.23	2.5
NM_001002236	A_17_P09484736	SERPINA1	chr14:093927140-093927185	non-CGI	449	221345.90	94985.24	2.3

NM_001002236	A_17_P09484738	SERPINA1	chr14:093927348-093927394	non-CGI	449	56231.88	21802.28	2.6
NM_024515	A_17_P09514219	MGC4645	chr14:099917321-099917367	non-CGI	347	142515.10	87295.66	1.6
NM_024515	A_17_P09514221	MGC4645	chr14:099917569-099917614	non-CGI	347	123798.80	63274.41	2.0
NM_002933	A_76_P0149154	RNASE1	chr14:20340559-20340619	non-CGI	885	22549.34	9537.50	2.4
NM_198235	A_76_P0138867	RNASE1	chr14:20341119-20341179	non-CGI	454	31263.73	21089.20	1.5
NM_015299	A_76_P0160969	KIAA0323	chr14:23967814-23967754	non-CGI	785	40568.48	13994.11	2.9
NM_014249	A_17_P09748983	NR2E3	chr15:069889880-069889932	non-CGI	95	7535.99	3188.58	2.4
NM_001013638	A_17_P09883529	LOC388199	chr16:000795533-000795578	non-CGI	891	71599.25	46143.25	1.6
NM_182563	A_17_P09889593	MGC21830	chr16:002201812-002201860	non-CGI	243	139117.20	79804.83	1.7
NM_133450	A_17_P09899494	KIAA1977	chr16:004720167-004720212	non-CGI	821	40131.45	24911.59	1.6
NM_033309	A_17_P10083340	MGC4655	chr16:065743274-065743319	non-CGI	897	65731.69	40691.05	1.6
NM_152456	A_17_P10096539	MGC34647	chr16:069238443-069238488	non-CGI	338	29280.82	16388.84	1.8
NM_152348	A_17_P10191174	FLJ33817	chr17:001576966-001577011	non-CGI	389	165377.30	65755.96	2.5
NM_152348	A_17_P10191176	FLJ33817	chr17:001577145-001577190	non-CGI	389	205586.20	82257.97	2.5
NM_152348	A_17_P10191178	FLJ33817	chr17:001577310-001577355	non-CGI	845	117401.40	67680.97	1.7
NM_152348	A_17_P10191179	FLJ33817	chr17:001577446-001577491	non-CGI	845	98013.57	41415.51	2.4
NM_152348	A_17_P10191181	FLJ33817	chr17:001577628-001577673	non-CGI	845	128098.20	57114.23	2.2
NM_152348	A_17_P10191183	FLJ33817	chr17:001577867-001577913	non-CGI	845	137144.20	67212.12	2.0
NM_152348	A_17_P10191185	FLJ33817	chr17:001577994-001578039	non-CGI	845	124895.70	59971.13	2.1
NM_152348	A_17_P10191187	FLJ33817	chr17:001578127-001578172	non-CGI	807	33401.64	18512.06	1.8
NM_152348	A_17_P10191188	FLJ33817	chr17:001578241-001578286	non-CGI	807	56087.40	25150.44	2.2
NM_145068	A_17_P10197862	TRPV3	chr17:003407727-003407772	non-CGI	541	109192.80	58109.47	1.9
NM_001671	A_17_P10212199	ASGR1	chr17:007023112-007023157	non-CGI	576	219931.20	105558.50	2.1
NM_000691	A_17_P10262154	ALDH3A1	chr17:019592484-019592529	non-CGI	276	158995.50	82659.04	1.9
NM_145110	A_17_P10265718	MAP2K3	chr17:021140991-021141039	non-CGI	789	192612.70	85528.02	2.3
NM_145110	A_17_P10265721	MAP2K3	chr17:021141278-021141323	non-CGI	789	125022.60	47442.46	2.6
NM_145110	A_17_P10265724	MAP2K3	chr17:021141546-021141591	non-CGI	789	175194.00	67604.88	2.6
NM_145110	A_17_P10265725	MAP2K3	chr17:021141708-021141753	non-CGI	130	38372.97	26149.19	1.5
NM_001015002	A_17_P10466810	LLGL2	chr17:071051337-071051389	non-CGI	907	117647.30	75158.59	1.6
NM_207389	A_17_P10491939	FLJ44861	chr17:076753015-076753060	non-CGI	805	77061.43	43380.86	1.8
NM_145068	A_76_P0159219	TRPV3	chr17:3407735-3407795	non-CGI	541	42882.21	16514.81	2.6
NM_004524	A_76_P0160658	LLGL2	chr17:71051540-71051600	non-CGI	907	50151.01	23396.12	2.1
NM_207389	A_76_P0140438	FLJ44861	chr17:76721061-76721001	non-CGI	129	12239.99	6919.87	1.8
NM_207389	A_76_P0162400	FLJ44861	chr17:76753268-76753208	non-CGI	805	19602.75	12318.58	1.6
NM_001611	A_17_P10884783	ACP5	chr19:011549111-011549156	non-CGI	167	67248.20	10920.79	6.2
NM_001611	A_17_P10884784	ACP5	chr19:011549264-011549309	non-CGI	387	177677.30	95560.67	1.9
NM_001611	A_17_P10884787	ACP5	chr19:011549485-011549537	non-CGI	387	185909.30	98980.06	1.9

NM_031941	A_17_P10901543	USHBP1	chr19:017236464-017236510	non-CGI	425	229532.80	155238.90	1.5
NM_007000	A_17_P10948513	UPK1A	chr19:040849595-040849641	non-CGI	787	123292.60	72713.60	1.7
NM_198125	A_17_P10949481	TYROBP	chr19:041091232-041091280	non-CGI	477	10140.81	6922.38	1.5
NM_033068	A_17_P10990581	ACPT	chr19:055985444-055985489	non-CGI	677	133782.60	29325.29	4.6
NM_199250	A_17_P10990628	MGC13170	chr19:055994191-055994236	non-CGI	394	201552.30	107125.50	1.9
NM_199250	A_17_P10990630	MGC13170	chr19:055994396-055994441	non-CGI	394	168035.50	87648.72	1.9
NM_024298	A_17_P10999406	LENG4	chr19:059369527-059369572	non-CGI	373	162373.30	92815.89	1.7
NM_024298	A_17_P10999408	LENG4	chr19:059369732-059369777	non-CGI	373	214380.20	116226.10	1.8
NM_020410	A_76_P0166318	ATP13A1	chr19:19621166-19621106	non-CGI	840	37278.38	24053.18	1.5
NM_020410	A_76_P0146545	ATP13A1	chr19:19621349-19621409	non-CGI	840	83403.66	50537.39	1.7
NM_020410	A_76_P0141526	ATP13A1	chr19:19621688-19621628	non-CGI	840	32173.67	15527.07	2.1
NM_007000	A_76_P0167906	UPK1A	chr19:40849529-40849469	non-CGI	787	61588.52	34002.62	1.8
NM_007000	A_76_P0149255	UPK1A	chr19:40849680-40849740	non-CGI	787	66281.05	34113.76	1.9
NM_172140	A_76_P0152429	IL29	chr19:44478560-44478620	non-CGI	855	25146.31	17306.01	1.5
NM_172140	A_76_P0168944	IL29	chr19:44478892-44478832	non-CGI	855	19913.39	12536.66	1.6
NM_172140	A_76_P0138600	IL29	chr19:44479111-44479171	non-CGI	855	28647.60	16262.84	1.8
NM_006114	A_76_P0160130	TOMM40	chr19:50082773-50082833	non-CGI	793	28454.68	16704.95	1.7
NM_033068	A_76_P0161378	ACPT	chr19:55985071-55985131	non-CGI	677	36599.01	9383.42	3.9
NM_033068	A_76_P0142949	ACPT	chr19:55985410-55985350	non-CGI	677	86579.83	27205.66	3.2
NM_182586	A_17_P00988277	FLJ33534	chr2:011222676-011222723	non-CGI	249	208846.10	103978.40	2.0
NM_080653	A_17_P01152721	ATP6V1E2	chr2:046657832-046657878	non-CGI	341	168126.70	55762.44	3.0
NM_144712	A_17_P01900464	SLC23A3	chr2:219859941-219859996	non-CGI	685	281883.90	89110.92	3.2
NM_144712	A_76_P0135717	SLC23A3	chr2:219860046-219860106	non-CGI	685	69824.07	32069.99	2.2
NM_144712	A_17_P01900466	SLC23A3	chr2:219860082-219860127	non-CGI	685	225585.30	81982.46	2.8
NM_001631	A_76_P0119215	ALPI	chr2:233145814-233145874	non-CGI	845	18802.22	9501.83	2.0
NM_001631	A_17_P01963021	ALPI	chr2:233145862-233145907	non-CGI	259	137456.60	78511.19	1.8
NM_001631	A_17_P01963023	ALPI	chr2:233146128-233146177	non-CGI	621	98862.70	58908.39	1.7
NM_001631	A_76_P0108171	ALPI	chr2:233146153-233146093	non-CGI	621	29233.83	19223.76	1.5
NM_001631	A_17_P01963024	ALPI	chr2:233146301-233146346	non-CGI	621	185335.80	89715.01	2.1
NM_001631	A_76_P0107821	ALPI	chr2:233146330-233146390	non-CGI	621	91549.58	53477.07	1.7
NM_001631	A_76_P0128731	ALPI	chr2:233146670-233146610	non-CGI	621	62294.43	29859.03	2.1
NM_003650	A_17_P11126228	CST7	chr20:024878151-024878196	non-CGI	108	63956.28	42605.03	1.5
NM_174897	A_17_P11136246	BPIL3	chr20:031082616-031082662	non-CGI	741	78682.82	52721.21	1.5
NM_174897	A_17_P11136252	BPIL3	chr20:031083179-031083224	non-CGI	741	101002.40	64034.33	1.6
NM_181467	A_17_P11144308	ITGB4BP	chr20:033336860-033336905	non-CGI	226	105870.30	72586.70	1.5
NM_003349	A_17_P11204511	Kua-UEV	chr20:048179938-048179983	non-CGI	301	77805.24	44163.06	1.8
NM_003650	A_76_P0151084	CST7	chr20:24878164-24878224	non-CGI	108	11049.83	5683.66	1.9

NM_174897	A_76_P0155234	BPIL3	chr20:31082791-31082731	non-CGI	741	58523.04	37241.21	1.6
NM_174897	A_76_P0138396	BPIL3	chr20:31082944-31083004	non-CGI	741	44553.22	26217.39	1.7
NM_174897	A_76_P0162663	BPIL3	chr20:31083231-31083171	non-CGI	741	46376.88	28411.70	1.6
NM_019601	A_17_P11445755	SUSD2	chr22:022901596-022901641	non-CGI	728	77996.01	33630.51	2.3
NM_019601	A_17_P11445756	SUSD2	chr22:022901727-022901772	non-CGI	728	49240.74	16620.20	3.0
NM_019601	A_17_P11445757	SUSD2	chr22:022901846-022901891	non-CGI	728	103668.20	33778.68	3.1
NM_001051	A_17_P11496472	SSTR3	chr22:035933394-035933439	non-CGI	832	43455.07	7287.93	6.0
NM_001051	A_17_P11496473	SSTR3	chr22:035933775-035933820	non-CGI	832	39165.25	5185.08	7.6
NM_025045	A_17_P11499956	BAIAP2L2	chr22:036830994-036831039	non-CGI	550	7155.76	4426.99	1.6
NM_019601	A_76_P0151644	SUSD2	chr22:22901456-22901396	non-CGI	728	33883.27	14650.97	2.3
NM_019601	A_76_P0146131	SUSD2	chr22:22901541-22901601	non-CGI	728	51577.48	17836.94	2.9
NM_019601	A_76_P0158758	SUSD2	chr22:22901871-22901811	non-CGI	728	53019.91	17376.13	3.1
NM_025045	A_76_P0171875	BAIAP2L2	chr22:36830861-36830801	non-CGI	550	16800.87	8562.55	2.0
NM_033199	A_17_P02222985	UCN2	chr3:048576350-048576397	non-CGI	915	40490.75	21443.08	1.9
NM_000094	A_17_P02222986	COL7A1	chr3:048576751-048576796	non-CGI	915	117077.00	58665.12	2.0
NM_003262	A_17_P02758680	TLOC1	chr3:171166382-171166442	non-CGI	460	38448.34	24774.39	1.6
NM_014965	A_76_P0101166	OIP106	chr3:42176114-42176054	non-CGI	771	5883.90	3861.95	1.5
NM_014965	A_76_P0102808	OIP106	chr3:42176334-42176394	non-CGI	771	24195.34	15268.97	1.6
NM_005847	A_17_P04318582	SLC23A1	chr5:138746661-138746720	non-CGI	652	20167.13	11422.59	1.8
NM_152685	A_17_P04318584	SLC23A1	chr5:138746903-138746960	non-CGI	652	19326.65	10583.85	1.8
NM_001264	A_17_P04642515	CDSN	chr6:031196222-031196267	non-CGI	971	34051.28	12040.19	2.8
NM_007109	A_76_P0105664	TCF19	chr6:31237000-31236940	non-CGI	955	39524.10	5682.83	7.0
NM_001013836	A_17_P05266161	MAD1L1	chr7:002042940-002042994	non-CGI	486	122089.00	60425.61	2.0
NM_001013836	A_17_P05266163	MAD1L1	chr7:002043120-002043172	non-CGI	486	97755.44	45411.87	2.2
NM_001013836	A_17_P05266165	MAD1L1	chr7:002043242-002043287	non-CGI	486	104818.60	46290.11	2.3
NM_000385	A_17_P05396989	AQP1	chr7:030723899-030723945	non-CGI	740	85587.13	54886.86	1.6
NM_000385	A_17_P05396991	AQP1	chr7:030724136-030724181	non-CGI	740	111672.80	73294.55	1.5
NM_006213	A_17_P05505786	PHKG1	chr7:055934828-055934885	non-CGI	135	104680.00	70752.33	1.5
NM_002360	A_76_P0116724	MAFK	chr7:1342598-1342538	non-CGI	149	49401.92	21019.69	2.4
NM_005775	A_17_P06006297	SCAM-1	chr8:022468109-022468154	non-CGI	381	382837.80	201370.20	1.9
NM_001013842	A_17_P06006545	LOC541565	chr8:022514554-022514599	non-CGI	710	15508.08	8235.56	1.9
NM_178828	A_17_P06801115	C9orf79	chr9:087727340-087727385	non-CGI	890	23869.79	5594.01	4.3
NM_000476	A_17_P06971105	AK1	chr9:127716130-127716175	non-CGI	642	296627.40	148694.00	2.0
NM_032874	A_17_P07010288	KIAA1984	chr9:136976660-136976705	non-CGI	583	373542.40	210014.90	1.8
NM_000954	A_17_P07011032	PTGDS	chr9:137144596-137144641	non-CGI	427	281192.50	177222.60	1.6
NM_000954	A_17_P07011033	PTGDS	chr9:137144741-137144786	non-CGI	427	181958.30	119273.00	1.5
NM_080877	A_17_P07012140	SLC34A3	chr9:137402226-137402271	non-CGI	543	230358.00	152788.40	1.5

NM_018969	A_17_P11751590	GPR173	chrX:052961420-052961465	non-CGI	526	70814.06	34834.70	2.0
NM_005032	A_17_P11902756	PLS3	chrX:114649878-114649938	non-CGI	408	44336.86	17685.37	2.5
NM_007150	A_17_P12031701	ZNF185	chrX:151757433-151757478	non-CGI	852	46847.97	22496.39	2.1
