# Supplementary material for: Genome-Wide Profiling of DNA Methylation Reveals a Class of Normally Methylated CpG Island Promoters
Source: PLoS Genet. 2007 Oct 26;3(10):e181. doi: 10.1371/journal.pgen.0030181 (PMC2041996; doi:10.1371/journal.pgen.0030181)
Supplement: Table S2 — (7 KB PDF) [file pgen.0030181.st002.pdf]

Supplementary Table 2. Validation of SssI treatment by methylation analysis of 41 CpG sites at 7 genes

Gene Name	No. of CG site	Methylation level (%)	
		before *SssI*	after *SssI*
**PEX3**	1	0	89
**PEX3**	2	3	91
**PEX3**	3	0	86
**PEX3**	4	0	91
**PEX3**	5	0	77
**PEX3**	6	0	86
**PEX3**	7	0	99
**UBQLN1**	8	1	96
**UBQLN1**	9	2	95
**UBQLN1**	10	0	74
**UBQLN1**	11	0	100
**UBQLN1**	12	1	98
**UBQLN1**	13	2	97
**UBQLN1**	14	2	98
**ABCB10**	15	0	83
**ABCB10**	16	0	67
**ABCB10**	17	0	96
**ABCB10**	18	0	71
**ABCB10**	19	0	94
**ABCB10**	20	0	91
**MPHOSPH10**	21	1	98
**MPHOSPH10**	22	0	98
**MPHOSPH10**	23	2	100
**MAP3K7**	24	18	90
**MAP3K7**	25	27	100
**MAP3K7**	26	0	92
**MAP3K7**	27	5	91
**MLL6**	28	3	88
**MLL6**	29	5	100
**MLL6**	30	0	84
**MLL6**	31	0	97
**MLL6**	32	3	100
**MLL6**	33	3	91
**PPAN**	34	4	92
**PPAN**	35	4	94
**PPAN**	36	0	100
**PPAN**	37	0	100
**PPAN**	38	0	92
**PPAN**	39	0	78
**PPAN**	40	4	95
**PPAN**	41	5	100
