# Supplementary material for: Genome-Wide Profiling of DNA Methylation Reveals a Class of Normally Methylated CpG Island Promoters
Source: PLoS Genet. 2007 Oct 26;3(10):e181. doi: 10.1371/journal.pgen.0030181 (PMC2041996; doi:10.1371/journal.pgen.0030181)
Supplement: Table S3 — (9 KB PDF) [file pgen.0030181.st003.pdf]

Supplementary Table 3. Primer sequences and PCR conditions for Bisulfit-pyrosequencing analysis

	PCR				Pyrosequencing	
**Gene**	**Formard primer**	**Reverse primer**	**Size (bp)**	**Annealing (C)**	**sequence primer**	**sequence region**
FANK1	GGGGATGAAGGGGTAAAGT	Biotin-Univ-ACCCTACCAACAACCCTC	208	55	AGTTAGGTTTATTGGGGA	YGatagYGaYGaYGgYGgYGgtt
ME1	TGTTAATAGTTTTGGGGATTTTT	Biotin-Univ-CCCTACCCRAAAAAACTAA	144	50	AATTTTTTTAGGTGTTTGAG	YGATYGTYGG TTTTGTTTGA ATYGTTTAGG TTYGTATTTT TTGGTATT
OPLAH	AAGYGGGAAGTGGGGTTTT	Biotin-Univ-AACRACCTAATACCCCCATAC	202	60	TTTTTTYGTGTTGAGGGAT	TTYGTTYGAGGGTTTTAGGGTTTAGYGGYGTTTTT
	AGAGTTTTTGTGGGATTTTAGTT	Biotin-Univ-TCCRCATAATTAACAAAATCCTCT	165	55	AGTTTTATTATTATGGGTAG	TTTYGAGGGTYGTTTTTATTTTGTTATYGATYGTGGGG
ANKRD30A	TGTTAAGYGGGTTATGTGGTATGG	Biotin-Univ-TATTCRCCAAACTCTTACACACT	226	50	GGTAGTTGTTATTTGTA	YGYGGTGTTYGGAGAAYGYGGTTAG
					TTTTTTAGTTTGTTGAGTTG	YGTATGYGTTGTTGGTTAAYGGTTTTGTTTAGYGYGATTTTA
	TTGAGAGAGGTTTGAGTGTGTAA	Biotin-Univ-ACAAAAATCTCCTCCATAACTAC			TGAGTGTGTAAGAGTTTGG	
	AGGTTTGAGTGTGTAAGAGTTTG	Biotin-Univ-TTTCTAAAATCCCCAAAATAAAC			TTATGGAGGAGATTTTTG	
INSL6	TTTAGGAGTTAGGGTTTGTTATTT	Biotin-Univ-CCCTATAACCCCAAACTAATC	228	55	TATTTGAGATTTTTGAGTTG	GYGGGAAAGTTTTGAYGTAGTYGGYGYGYGTTTTTTTTTGA
					TTTTTGAAGTTTTGG	YGAYGTTTTTAYGYGttYGgaatttYGtagtgtttgt
					GTAGATAGGGAGTAGGGTT	YGgtYGatYGttattgtataaYGYGgag
FLJ40201	AGGGAAGGATTTTGAAAATGT	Biotin-Univ-CATCCTAACTTTCCCTCATAACAA	186	60	GGATAAGTTAAAAATTAG	YGggttaYGtgYGtaYGtYGgtggggg
MEST	AGGTTTTGATGATAGGTTTATAGG	Biotin-Univ-RATACCCRCTACATCCAAA	188	55	GYGGTAGTTTTTTTAGTGTT	YGtgggtYGttttYGgt*ttYGgg* ttggatgtttYGYGgtttagtat
FTMT	GGGGYGTTTTTTGGATTTTAG	Biotin-Univ-CCCRAAAAAAATAATAAACCATA	209	60	TTTTTGGATTTTAGGTAGAT	YGTTTTTYGTYGTTTTTTGGTYGTAGTYGTTTTTT *TTYGGG* ATTTTAT
KCNE4	GTGGAGGTAGTTTTAATGTTGAA	Biotin-Univ-TACCRTAAAAAAACATAACAACC	157	50	GAAAATGGAGTTTTTGAATA	GTAYGTATTTYGGTATYGTYGTTTTTAGTAGTTTT
					TTAGTAGTTTTTTGGAGTTT	YGTGYGGTYGGYGGYGGTAGYGGTAATGGT
SOHLH2	TGTTTTGGGAAGGGGATTT	Biotin-Univ-CCCCAAACRCCCTTAAAATA	187	55	AGGATAGTTTGAGTAGTTTT	YGYGGGTAGGTTTTYGATTYGGGTTTYGYGTTGATTT
	TTGGYGATAGGGTTATTTTAAGG	Biotin-Univ-CTAACCCRAAATCTAACAATACTC	201	55	AGGAGTTATTAGTGTAGGGG	gYGggttYGggaYGtagYGgttgtggagtttttttgttg
					GGTTGTTTTAGGATTTAGTA	YGTGYGTAGYGGTTAT
ZNF541	GGGTTGTTTTAAAAGTAAAGGTAA	Biotin-Univ-AAAACTTCCRCTACTAAATCCC	109	55	GTGTTTGTTGTTTATAAGTT	TTYGGTYGTGTTTTYGYGGGAGGGTTTYGAGT
	TAGYGGGGAAGATGATTTTTG	Biotin-Univ-AAAATCCRCTAAAACTTAATAATC	228	55	GTTTTTAAGAAGGTTAAG	GTYGATTGYGATTTTTttttgtgttagaatttYGggagtt
					TAGGAGGTTTAGAAGGTAGG	YGggtttttgYGgatgtttYGtYGtttttYGttagttttttt
SHANK1	ATGGTYGTTTTTGGTTTTTGTAG	Biotin-Univ-CCTAACCCRTCTAAAAAACTATC	167	60	TTTATAATGATTTATAGTTT	YGYGATAAGYGAGGAYGAGGAAYGTTATAGTG
RNF113B	TGTTTYGGGATTATAAGAGTGAGT	Biotin-Univ-AACRCTACCATATTTAAATCTCAA	177	60	TTAGAATTGGGAGGGTT	taYGttttaYGtgtagYGaYGtaagggYGaYGtaaggg
CTL2	GGYGTTGTGTTGGGAGGAGT	Biotin-Univ-TCRTCCCCCATCCCTACA	131		TTAGTGTTTTTTTTTAGATT	YGGGAGGGTYGAGGGGGYGYGGGAGAG
ACTL7B	TGAAGTTTAGGAAGGTGTATAAGA	Biotin-Univ-ACTTCAACRAATTCACCAACTTAA			TTGGGTTTTTAGTATTGTAA	GTGYGGTTAYGYGGGAGAGTYGAGG
					TGGGTTATGAGTTGTTTAAT	AYGGAGGYGTTTT
DPPA5	GGTTAATGTAGGTGGAAATTTTA	Biotin-Univ-ACCAAAACCCTAAATCCATAC	140	50	AATTTGGAGATAGTTGGGTT	YGGGTGAGGTTTTTTYGAAGTYGYGGGAAGYGGAAGA
					GGGTGAGGTTTTTT	YGAAGTYGYGGGAAGYGGAAG
					GAGTTGGTAATTTGTTGGAT	YGTAGTTAGGGYGGYGGGGT
C19orf24	GGGAAGATTGTAAGGGTTATTGTT	Biotin-Univ-CTCCCAAACCTACTACAAAAAAC	189	55	GTTATTGAGGGGGT	TYGTAGYGYGGAGATTTTAGTTTYGYGGGATTTG
C12orf12	GATAGTGAAGTATTTGGGTTGAGA	Biotin-Univ-ACRCAATAAAACCCACTCC	125	60	GGGTGTGGTGGGGAT	TYGGGAGTGG GTTTTATTG
					GAGGGAGTAGTAGGTAATTG	YGTAATTTTTGTTTTTTTYGGTTTTAGTTYGT
	TTGTGGTTGTGGTTGGGTATA	Biotin-Univ-AACACCCCCAATTAAAACACAC	200	60	TGGGTTTTTAAGGAAGTAG	TYGAAGTAAT AGTAYGGTTY GGGTTTTTGG TTTTAATTAT T
					TTTTGAGTTTTTGGT	YGTTTTGTTATYGAAGGYGTTYGGGTGT
PFN2	GGGTAGYGAATATAGGAGAAAAGT	Biotin-Univ-CRACRAAAAAAAATAAACCC	115	60	ATATAGGAGAAAAGTATAT	YGYGGTTYGGTYGYGGGGTTTTAA
