# Supplementary material for: Genome-Wide Profiling of DNA Methylation Reveals a Class of Normally Methylated CpG Island Promoters
Source: PLoS Genet. 2007 Oct 26;3(10):e181. doi: 10.1371/journal.pgen.0030181 (PMC2041996; doi:10.1371/journal.pgen.0030181)
Supplement: Table S4 — (7 KB PDF) [file pgen.0030181.st004.pdf]

Supplementary Table 4. Primer sequences and PCR conditions for RT-PCR analysis

		RT-PCR					
**Gene**	**Accession Number**	**Formard primer (5'-3')**	**Reverse primer (5'-3')**	**Size (bp)**	**Annealing (C)**	**cycles**	**exons analyzed**
ANKRD30A	NM_052997	CAAAGAGGAAGAAGACCATCAACC	TGATGGCATTGTAGAGCCTTCAT	181	55	35	ex1-3
INSL6	NM_007179	AAAAACTCTGCGGCCATGC	CCCAACTGTTTACTGCTTCTTCC	201	55	35	ex1-2
FLJ40201	NM_152607	TGCAGAGCCACAGGTTTGAC	CCTGTGTCCACCTCCTTCATC	181	55	35	ex2-3
SOHLH2	NM_017826.1	TATCTGCCAGGAGCACTGCC	GAATATGCAATCATCCAAAAGCG	187	55	35	ex1-2
DPPA5	NM_001025290	GGAACTCTCCCGGCACGTA	CGTAAACCACGACCTCGGTG	211	55	35	ex1-2
GAPDH	AF261085	TCCCATCACCATCTTCCAG	ATGAGTCCTTCCACGATACC	309	50	25	NA
